# Functional connectivity across the human subcortical auditory system using an autoregressive matrix-Gaussian copula graphical model approach with partial correlations

**DOI:** 10.1162/imag_a_00258

**Published:** 2024-08-12

**Authors:** Noirrit Kiran Chandra, Kevin R. Sitek, Bharath Chandrasekaran, Abhra Sarkar

**Affiliations:** The University of Texas at Dallas, Department of Mathematical Sciences, Richardson, TX 76010, USA; Northwestern University, Roxelyn and Richard Pepper Department of Communication Sciences and Disorders, Evanston, IL 60208, USA; The University of Texas at Austin, Department of Statistics and Data Sciences, Austin, TX 78712, USA

**Keywords:** functional MRI, Gaussian copula graphical model, partial correlation, resting-state connectivity, subcortical auditory system, timeseries

## Abstract

The auditory system comprises multiple subcortical brain structures that process and refine incoming acoustic signals along the primary auditory pathway. Due to technical limitations of imaging small structures deep inside the brain, most of our knowledge of the subcortical auditory system is based on research in animal models using invasive methodologies. Advances in ultrahigh-field functional magnetic resonance imaging (fMRI) acquisition have enabled novel noninvasive investigations of the human auditory subcortex, including fundamental features of auditory representation such as tonotopy and periodotopy. However, functional connectivity across subcortical networks is still underexplored in humans, with ongoing development of related methods. Traditionally, functional connectivity is estimated from fMRI data with full correlation matrices. However, partial correlations reveal the relationship between two regions after removing the effects of all other regions, reflecting more direct connectivity. Partial correlation analysis is particularly promising in the ascending auditory system, where sensory information is passed in an obligatory manner, from nucleus to nucleus up the primary auditory pathway, providing redundant but also increasingly abstract representations of auditory stimuli. While most existing methods for learning conditional dependency structures based on partial correlations assume independently and identically Gaussian distributed data, fMRI data exhibit significant deviations from Gaussianity as well as high-temporal autocorrelation. In this paper, we developed an autoregressive matrix-Gaussian copula graphical model (ARMGCGM) approach to estimate the partial correlations and thereby infer the functional connectivity patterns within the auditory system while appropriately accounting for autocorrelations between successive fMRI scans. Our results show strong positive partial correlations between successive structures in the primary auditory pathway on each side (left and right), including between auditory midbrain and thalamus, and between primary and associative auditory cortex. These results are highly stable when splitting the data in halves according to the acquisition schemes and computing partial correlations separately for each half of the data, as well as across cross-validation folds. In contrast, full correlation-based analysis identified a rich network of interconnectivity that was not specific to adjacent nodes along the pathway. Overall, our results demonstrate that unique functional connectivity patterns along the auditory pathway are recoverable using novel connectivity approaches and that our connectivity methods are reliable across multiple acquisitions.

## 1 Introduction

The mammalian auditory pathway conveys acoustic information between the inner ear—where sounds are mechanoelectrically transduced in the cochlea—and auditory cortex by way of multiple subcortical nuclei across the brainstem, midbrain, and thalamus. Much of our knowledge of the auditory system arises from anatomical and physiological research with nonhuman animal models (Brugge, 1992;McIntosh & Gonzalez-Lima, 1991;Webster, 1992). This work has contributed tremendously to our understanding of the mammalian auditory system. However, due to methodological and ethical limitations, our ability to directly assess auditory function in the human nervous system is severely constrained (Moerel et al., 2021).

This is particularly true for subcortical auditory structures. In mammals, the ascending central auditory pathway receives signals from the cochlea of the inner ear by way of the cochlear nerve, which principally innervates the cochlear nucleus in the brainstem. Auditory signals are then transmitted to the superior olivary complex, which is the first decussation point at which signals largely pass contralaterally from the left cochlear nucleus to the right superior olive (and similarly from right to left). From the superior olive, auditory signals travel (via the lateral lemniscus) to the inferior colliculus in the midbrain. The last subcortical auditory structure is the medial geniculate nucleus of the thalamus, which then passes information to the primary auditory cortex. In addition to the ascending “lemniscal” auditory pathway, an equal number of efferent connections transmit top-down information from higher order auditory regions to earlier auditory structures (Malmierca & Ryugo, 2011;Winer, 2005). Due to the small size of the subcortical auditory structures—tightly packed in with other heterogeneous nuclei and white matter pathways—and their anatomical location deep within the cranium, the subcortical auditory structures have received limited attention in noninvasive human research.

Functional magnetic resonance imaging (fMRI) is the most popular noninvasive method for probing macroscopic network-related brain activity. While studies of the human subcortical auditory system are somewhat limited, previous task-based fMRI research has functionally localized the subcortical auditory structures (Sitek et al., 2019), identified the tonotopic frequency mappings within the auditory midbrain and thalamus (De Martino et al., 2013;Moerel et al., 2015;Ress & Chandrasekaran, 2013), separated top-down and bottom-up speech-selective subregions of auditory thalamus (Mihai et al., 2019;Tabas et al., 2021), and recorded level-dependent BOLD signals throughout the auditory pathway (Hawley et al., 2005;Sigalovsky & Melcher, 2006).

In contrast to task-related BOLD activity, fMRI connectivity methods (often utilizing resting-state fMRI paradigms and full correlation analysis) are commonly used to assay cortical brain networks (Biswal et al., 1995;Gordon, Laumann, Adeyemo, et al., 2017;Power et al., 2011;Smith, Beckmann, et al., 2013), including the cortical auditory system (Abrams et al., 2020;Cha et al., 2016;Chen et al., 2020;Eckert et al., 2008;Maudoux et al., 2012;Ren et al., 2021). However, fMRI connectivity methods have limited history in subcortical research, especially in the auditory system, where they have—to our knowledge—only been utilized a handful of times to assess connectivity differences between individuals with and without tinnitus percepts (Berlot et al., 2020;Hofmeier et al., 2018;Leaver et al., 2016;J. Zhang et al., 2015).

From the seminal resting-state connectivity studies identifying human default mode and motor networks (Biswal et al., 1995;Fox & Raichle, 2007), to work linking functional connectivity with behavioral variability (Baldassarre et al., 2012;Deng et al., 2016), to investigations into brain network differences associated with disorders (Chai et al., 2016;Di Martino et al., 2011;Ferri et al., 2018;Greicius et al., 2007;Hahn et al., 2011;Husain & Schmidt, 2014;Kaiser et al., 2015;Sitek et al., 2016;Wilson et al., 2022), full correlation analysis has contributed tremendously to our understanding of human brain networks. However, moving beyond the traditional full correlation analysis should enable greater specificity in assessing functional connectivity patterns, particularly in identifying specific node-to-node connectivity patterns within an established brain network (Marrelec et al., 2006;Smith, 2012). In contrast to full correlations, which represent both direct and indirect connections, partial correlation analyses represent the direct association between two specific nodes after filtering out the effects of the remaining nodes and thus hold great promise for estimating direct functional connectivity within a network (Smith, Beckmann, et al., 2013). (Refer to the illustration in Section S1.1 in theSupplementary Materials.) For these reasons, partial correlation approaches are increasingly used to study functional connectivity networks in the brain (Wang et al., 2016;Warnick et al., 2018), including improved identification of individualized connectivity profiles compared with full correlation methods (Menon & Krishnamurthy, 2019), as well as improved prediction of brain disorders (de Vos et al., 2018;Reeves et al., 2023;Skåtun et al., 2017) and identification of general cognitive ability (Sripada et al., 2021). However, we are unaware of the application of such methods to assess functional connectivity within subcortical networks, particularly within the human auditory system.

In this article, we build on the Bayesian precision factor model (PFM) introduced recently inChandra et al. (2021)to develop a novel highly robust autoregressive matrix-Gaussian copula graphical model (ARMGCGM) to assess partial correlation-based functional connectivity in a specific network in the human brain that spans subcortical and cortical regions, the auditory system. Notably, partial correlations can be readily obtained from the precision matrix, i.e., the inverse of the covariance matrix. The PFM decomposes the model precision matrix into a flexible low-rank and diagonal structure, then exploits that to design efficient estimation algorithms. However, PFM makes the assumption that data are independent and identically distributed (iid) multivariate Gaussian random variables. Several studies in the literature also make this assumption (Yu et al., 2022;L. Zhang et al., 2014) which has the very useful implication that a zero partial correlation between two variables (equivalent to a zero entry in the precision matrix in the corresponding position) also means independence between them after removing the effects of other variables. However, in many applications—including ours—the iid Gaussian assumption can be restrictive as data are often not Gaussian distributed. Additionally, data from successive fMRI volumes exhibit strong autocorrelation. The ARMGCGM extends the PFM to the case where the univariate marginals can be any arbitrary distribution while also accounting for the autocorrelations between successive fMRI scans. The association between the variables is modeled using a Gaussian copula that implies conditional independence for zero partial correlation, allowing easy interpretability of the conditional dependence graph. As developed, the ARMGCGM approach is broadly applicable for studying undirected functional graphs using large-scale fMRI data.

We use the novel ARMGCGM to investigate functional connectivity between specific nodes across the human auditory system. We used publicly available 7T resting-state fMRI from over 100 participants to examine auditory connectivity. To probe connectivity within the auditory system, we included auditory cortical regions of interest as well as subcortical auditory regions derived from human histology (Sitek et al., 2019). As the auditory pathway comprises a chain of multiple subcortical structures, and due to the largely lateralized organization of the lemniscal auditory system, we hypothesized that partial correlations would be greatest between adjacent nodes in the same hemisphere, over and above the contributions from other auditory (and nonauditory) regions of interest. In particular, because of the critical position of auditory midbrain and thalamus as computational hubs involving bottom-up and top-down information transfer (Mihai et al., 2019;Tabas et al., 2021), we hypothesized strong partial correlations between inferior colliculus and medial geniculate. We further assessed reliability across acquisitions by separately analyzing data with anterior–posterior and posterior–anterior phase-encoding directions, as well as leave-10%-out cross-validation. We then compared our ARMGCGM-based connectivity results with those from a full correlation approach as well as alternative partial correlation methods. Overall, our consistent findings of hierarchical connectivity within the auditory system using our novel partial correlation method—consistent across data partitions and leave-10%-out validation— demonstrate the methodological reliability of our ARMGCGM approach as well as the neurobiological organization of auditory structures in the human primary auditory system.

## 2 Materials and Methods

### 2.1 Magnetic resonance imaging acquisition and processing

We used resting-state fMRI from 106 participants in the 7T Human Connectome Project (Elam et al., 2021;Van Essen et al., 2012). Specifically, we utilized the minimally preprocessed volumetric data in common space (Glasser et al., 2013). BOLD fMRI data were acquired with 1.6 mm isotropic voxel size across four runs of a resting-state paradigm (repetition time [TR] = 1 s, 900 TRs per run). Two runs were acquired with anterior–posterior (AP) phase encoding, and two were acquired with posterior–anterior (PA) phase encoding. For all regions of interest (ROIs) and runs, we discarded the first 50 TRs to increase stability.

For each individual and each run, we extracted mean timeseries from predefined regions of interest (ROIs). Subcortical auditory ROIs were defined using the Sitek–Gulban atlas (Sitek et al., 2019). Cortical ROIs were defined using FreeSurfer’s implementation of the DKT atlas (Dale et al., 1999;Klein & Tourville, 2012). For this study we used transverse temporal gyrus (TTG) and superior temporal gyrus (STG) as auditory ROIs, as well as pericalcarine cortex (Calc) and superior frontal gyrus (SFG) as nonauditory control ROIs (see Section S1.5 and Fig. S3 inSupplementary Materials). Mean timeseries were extracted for each ROI using Nilearn’s [Masker] function. For an overview of materials and methods used in this study, please see Fig. S4 inSupplementary MaterialsSection 1.6.

### 2.2 Data partitioning and cross-validation

BOLD fMRI is prone to geometric distortions in the phase-encoding direction which can be largely corrected using a variety of methods (Esteban et al., 2021;Jezzard & Balaban, 1995). Adjacent to motion-sensitive cerebrospinal fluid (CSF), the brainstem is particularly susceptible to such geometric distortions. Although the HCP minimal preprocessing pipeline corrects for phase-encoding distortions (Glasser et al., 2013), to isolate the potential residual contribution of phase-encoding direction on functional connectivity estimates, we conducted separate analyses on data collected with the posterior–anterior (PA) phase-encoding direction (runs 1 and 3) and the anterior–posterior (AP) phase-encoding direction (runs 2 and 4) and compared the results. As the fMRI data acquired in the two phases will be analyzed separately but using the same probability-model, we use the same notations for the different phases to describe our proposed model in the following sections.

We further evaluated our results using a leave-10%-out cross-validation (CV) approach. We assessed the stability of the connectivity estimates by checking the correlation between the results obtained from each acquisition scheme.

### 2.3 Autoregressive matrix-Gaussian copula graphical models

The Bayesian precision factor model (PFM), developed recently inChandra et al. (2021), provides a novel computationally efficient robust technique for estimating precision matrices. Since partial correlations can be readily obtained from the precision matrix, the approach allows straightforward estimation of the underlying connectivity graphs. A plausible alternative could be to obtain the precision matrix by modeling the covariance matrix and then inverting its estimate (Lee & Kim, 2021). However, this approach often tends to exhibit poor empirical performance (Pourahmadi, 2013) and hence direct estimation of the precision matrix, if possible, may be preferable.

Most partial correlation-based conditional dependency graph estimation procedures in the statistical literature (Cai et al., 2020;Chandra et al., 2021;Friedman et al., 2008;Warnick et al., 2018) assume that the joint distribution of the data is the independent and identically distributed (iid) multivariate Gaussian, which implies that the univariate marginal distributions are also all Gaussians. However, successive fMRI scans have strong autocorrelations, and the marginal distributions for different ROIs exhibit substantial deviance from the Gaussian assumption. In this paper, we, therefore, extend the PFM to accommodate non-Gaussian marginals, while also appropriately accounting for their temporal dependence between successive scans.

Let${\left\{Y_{t,\;j}^{\left(r,i\right)}\right\}}_{t=1}^{T}$be the fMRI timeseries corresponding to thei-th individual’sj-th ROI at thet-th time point in ther-th run. In our application we are interested in studying the connectivity betweend=12ROIs along the central auditory pathway using fMRI timeseries of lengthT=850fromN=106individuals each undergoingR=2runs. We let$f_{j}^{\left(r,i\right)}$be the (unknown) marginal density of$Y_{t,\;j}^{\left(r,i\right)}$with corresponding cumulative distribution function (CDF)$F_{j}^{\left(r,i\right)}$.

Copulas provide a broadly applicable class of tools that allow the joint distribution of$Y_{t,j}^{\left(r,i\right)}$to be flexibly characterized by first modeling the univariate marginals$f_{j}^{\left(r,i\right)}$and then hierarchically modeling their dependencies by mapping the$F_{j}^{\left(r,i\right)}\left(Y_{t,\;j}^{\left(r,i\right)}\right)$’s to a joint probability space. For Gaussian copulas, this is done by setting$Z_{t,j}^{\left(r,i\right)}=\Phi^{-1}\left\{F_{j}^{\left(r,i\right)}\left(Y_{t,j}^{\left(r,i\right)}\right)\right\}$, whereΦ( ⋅ )is the CDF of a standard Gaussian distribution. This implies marginally$Z_{t,j}^{\left(r,i\right)}∼\text{N(0,1)}$for allr,i,j,t, where$\text{N(}\mu ,\sigma^{2}\text{)}$denotes a univariate Gaussian distribution with meanμand variance$\sigma^{2}$. We let${\text{Z}}^{\left(r,i\right)}={\left(\left(Z_{t,j}^{\left(r,i\right)}\right)\right)}_{T\times d}$denote the matrix of fMRI signals corresponding to thei-th individual in ther-th run in the transformed Gaussian space. The Gaussian copula assumption on$F_{t,j}^{\left(r,i\right)}\left(Y_{t,j}^{\left(r,i\right)}\right)$’s implies that the joint distribution of${\text{Z}}^{\left(r,i\right)}$is Gaussian as well. The dependencies between the$Z_{t,j}^{\left(r,i\right)}$’s are, therefore, characterized entirely by their correlations. Additionally, since the dependence relationships between the observed$Y_{t,j}^{\left(r,i\right)}$’s are modeled only through$Z_{t,j}^{\left(r,i\right)}$’s, these correlations also completely characterize the dependencies between the$Y_{t,j}^{\left(r,i\right)}$’s.

Note that probabilistic dependencies exist between$Z_{t,j}^{\left(r,i\right)}$’s across bothjandt; dependence acrossjincurs due to the interaction between the ROIs, whereas dependence acrosstoccurs due to the temporal dependence between successive fMRI scans. We let${\text{R}}_{\text{Ω}}$denote thed×dcorrelation matrix accounting for the dependence across theddifferent ROIs across all runs and individuals. While our main interest lies in estimating these dependencies between the ROIs, it is also crucial to consider the temporal dependence in the$Y_{t,j}^{\left(r,i\right)}$’s. Let${\text{Z}}_{.,j}^{\left(r,i\right)}={\left(Z_{1,j}^{\left(r,i\right)},\ldots ,Z_{T,j}^{\left(r,i\right)}\right)}^{T}$be thej-th column of${\text{Z}}^{\left(r,i\right)}$, i.e., the timeseries corresponding to thei-th individual’sj-th ROI in ther-th run.

We develop our model in a hierarchical manner. To begin with, we use autoregressive (AR) processes of orderLto model higher-order temporal dependencies in the$Z_{t,j}^{\left(r,i\right)}$’s as

$$\begin{array}{l} Z_{t,j}^{\left(r,i\right)}=\sum_{t^{\prime }=1}^{L}\beta_{t^{\prime },j}^{\left(r,i\right)}Z_{t-t^{\prime },j}^{\left(r,i\right)}+\in_{t,j}^{\left(r,i\right)},\;\;\;\;\;\;\;\;\in_{1,j}^{\left(r,i\right)},\ldots ,\\ \;\;\;\;\;\;\;\;\;\;\;\;\;\;\in_{T,j}^{\left(r,i\right)}\overset{iid}{∼}N\left(0,\varsigma_{j}^{2(r,i)}\right), \end{array}$$ (1)

with$Z_{t-t^{\prime },j}^{\left(r,i\right)}=0$if*$t^{\prime }\geq t$.*We assume separate$\left(\beta ,\varsigma^{2}\right)$parameters across(r,i,j)inequation (1)to make the model adapt to different timeseries patterns across different ROIs, individuals, and runs. Let$\xi_{.,j}^{\left(r,i\right)}$be the standardized AR-corrected timeseries corresponding to thej-th ROI forj=1,…,d. Define theT×dmatrix$\Xi^{\left(r,i\right)}=\left(\begin{array}{ccc} \xi_{\cdot ,1}^{\left(r,i\right)} & \ldots & \xi_{\cdot ,d}^{\left(r,i\right)} \end{array}\right)={\left(\begin{array}{ccc} \xi_{1,\cdot }^{\left(r,i\right)} & \ldots & \xi_{T,\cdot }^{\left(r,i\right)} \end{array}\right)}^{T}$where$\xi_{t,\cdot }^{\left(r,i\right)}$is thet-th row of$\Xi^{\left(r,i\right)}$and can be interpreted as the fMRI signals in the Gaussian copula space subsequent to filtering out the temporal dependence at time pointt. We then let

$$\xi_{1,\cdot }^{\left(r,i\right)},\ldots ,\xi_{T,\cdot }^{\left(r,i\right)}\overset{\text{iid}}{∼}\text{N}\left(\text{0},{\text{R}}_{\text{Ω}}\right).$$ (2)

From the property of Gaussian copula, the probabilistic dependence structure between the ROIs is entirely characterized by the correlation matrix${\text{R}}_{\Omega }$. This strategy thus allows borrowing of information across subjects as well as runs in estimating the common correlation matrix${\text{R}}_{\Omega }$resulting in improved statistical precision. The formulations inequations (1)-(2)imply the following joint distribution on${\text{Z}}^{\left(r,i\right)}$

$$f\left({\text{Z}}^{\left(r,i\right)}|{\text{R}}_{\text{Ω}},{\text{R}}_{T,1}^{\left(r,i\right)},\ldots ,{\text{R}}_{T,d}^{\left(r,i\right)}\right)=\frac{e^{-\frac{1}{2}\text{tr}\left({\text{R}}_{\Omega }^{-1}\Xi^{(r,i)T}\Xi^{\left(r,i\right)}\right)}}{{\left(2\text{π}\right)}^{\frac{dT}{2}}{\left|{\text{R}}_{\text{Ω}}\right|}^{\frac{T}{2}}\prod_{j=1}^{d}{\left|{\text{R}}_{T,j}^{\left(r,i\right)}\right|}^{\frac{1}{2}}},$$ (3)

where${\text{R}}_{T,\;j}^{\left(r,i\right)}$is the correlation matrix of${\text{Z}}_{\cdot ,j}^{\left(r,i\right)}$implied by theAR(L)model inequation (1).

Note that while the AR-corrected timeseries$\xi_{\cdot ,j}^{\left(r,i\right)}$’s are required to implement the model using MCMC, they can be obtained from the autoregressive parameters$\left\{\beta_{1,j}^{\left(r,i\right)},\ldots ,\beta_{L,j}^{\left(r,i\right)},\varsigma_{j}^{2(r,i)}\right\}$without having to invert the massive(T−1)×(T−1)dimensional matrices${\text{R}}_{T,j}^{\left(r,i\right)}$. Exploiting the conditionally linear structure fromequation (1), the$\xi_{\cdot ,j}^{\left(r,i\right)}$’s can be obtained without actually factorizing or inverting the${\text{R}}_{T,j}^{\left(r,i\right)}$’s. Avoiding such expensive matrix inversion operations makes our model implementation highly scalable; we discuss the details in Section S1.2 ofSupplementary Materials.

Next, let$\text{Ω}=\left(\left(\omega_{j,j^{\prime }}\right)\right)$be a precision matrix corresponding to${\text{R}}_{\text{Ω}}$, i.e.,$R_{\Omega }=\Psi^{-\frac{1}{2}}\Omega^{-1}\Psi^{-\frac{1}{2}}$. with$\Psi =\text{diag(}{\text{Ω}}^{-1}\text{)}$. Then, by properties of copula and the simple multivariate Gaussian distribution, it can be shown that, irrespective of the form of the marginal$f_{j}^{\left(r,i\right)}$’s, the ROIs$Y_{t,j}^{\left(r,i\right)}$and$Y_{t,j^{\prime }}^{\left(r,i\right)}$will be conditionally dependent on (and hence functionally connected with) each other given the rest if and only if$\omega_{j,j^{\prime }}\neq 0$. This way, the precision matrixΩcharacterizes the functional connectivity between the different$Y_{t,j}^{\left(r,i\right)}$’s acrossj.

To estimateΩ, we consider the framework ofChandra et al. (2021). We assumeΩto admit a lower-rank plus diagonal (LRD) decomposition$\Omega =\Lambda \Lambda^{T}+\Delta$whereΛis ad×qmatrix and a diagonal matrix$\Delta =\text{diag}\left(\delta_{1}^{2},\ldots ,\delta_{d}^{2}\right)$with positive$\delta_{j}^{2}$’s. Note that all positive definite matrices admit such a representation for someq≤d.

Finally, we model the unknown univariate marginal distributions$f_{j}^{\left(r,i\right)}$’s using location-scale mixtures of Gaussians. Such mixtures can flexibly approximate a very large class of densities (Ghosal et al., 1999). Specifically, we let

$$f_{j}^{\left(r,i\right)}=\sum_{h=1}^{K}\pi_{h,j}^{\left(r,i\right)}N\left(\mu_{h,j}^{\left(r,i\right)},\sigma_{h,j}^{2(r,i)}\right),$$ (4)

where${\text{π}}_{h,j}^{\left(r,i\right)}$is the weight attached to theh-th mixture component and$\sum_{h=1}^{K}{\text{π}}_{h,j}^{\left(r,i\right)}=1$withKbeing a suitably chosen, moderately large, fixed integer.

While fMRI timeseries often showcase very distinct patterns and distributions for different individuals as well as across different ROIs of the same individual, we expect primary sensory region to be highly similar between healthy participants due to strongly stereotyped processing across individuals and well-conserved auditory function in these brain regions across evolution (Hutchison et al., 2013). Inequation (4), we, therefore, consider separate mixture models across different ROIs, individuals, and runs, whereas inequation (3), the${\text{Z}}^{\left(r,i\right)}$’s share a common correlation matrix${\text{R}}_{\text{Ω}}$across all individuals and runs. This modeling strategy also allows borrowing of information across individuals and runs to amplify signals for estimating resting-state functional connectivity in adult humans while accounting for subject and run-specific variabilities using separate marginal distributions. This is conceptually similar to approaches used in group independent component analysis (Calhoun et al., 2001) and cohort-level brain mapping (Varoquaux et al., 2013). In later sections, we showed that this approach yields highly consistent estimates of functional connectivity graphs even though BOLD signals from small deep brain regions have very low signal-to-noise ratio (Bianciardi et al., 2016;Colizoli et al., 2020;de Hollander et al., 2017;Sclocco et al., 2018).

#### 2.3.1 Summary of the ARMGCGM

With fMRI timeseries data${\left\{Y_{t,j}^{\left(r,i\right)}\right\}}_{t=1}^{T}$corresponding to thei-th individual’sj-th ROI at thet-th time point in ther-th run, our hierarchical approach can be summarized as the following:

- The marginal densities$f_{j}^{\left(r,i\right)}$of$Y_{1,j}^{\left(r,i\right)},\ldots ,Y_{T,j}^{\left(r,i\right)}$are modeled using flexible location-scale mixtures of univariate Gaussians as specified inequation (4).
- Letting$F_{j}^{\left(r,i\right)}$be the corresponding CDF of$Y_{1,j}^{\left(r,i\right)},\ldots ,Y_{T,j}^{\left(r,i\right)}$, the timeseries are transformed into a Gaussian space$Z_{t,j}^{\left(r,i\right)}=\Phi^{-1}\left\{F_{j}^{\left(r,i\right)}\left(Y_{t,j}^{\left(r,i\right)}\right)\right\}$implying that marginally$Z_{t,j}^{\left(r,i\right)}∼\text{N}\left(\text{0,1}\right)$for allr,i,j,t.
- Autocorrelation-corrected$\xi_{t,j}^{\left(r,i\right)}$’s are obtained from the$Z_{t,j}^{\left(r,i\right)}$’s using theAR(L)model fromequation (1). Accordingly, the$\xi_{t,.}^{\left(r,i\right)}={\left(\xi_{t,1},\ldots ,\xi_{t,d}\right)}^{T}\prime s$are iid$\text{N}\left(\text{0},{\text{R}}_{\Omega }\right)$random vectors across allr,i,t.
- Let${\text{R}}_{\text{Ω}}=\Psi^{-\frac{1}{2}}{\text{Ω}}^{-1}\Psi^{-\frac{1}{2}}$with$\Psi =\text{diag}\left({\text{Ω}}^{-1}\right)$, whereΩis the precision matrix of the$\xi_{t,.}^{\left(r,i\right)}$’s. The precision factor model framework ofChandra et al. (2021)is then used to estimateΩ.

InFigure 1we provide a graphical illustration of the hierarchical structure of the ARMGCGM.

**Fig. 1.**
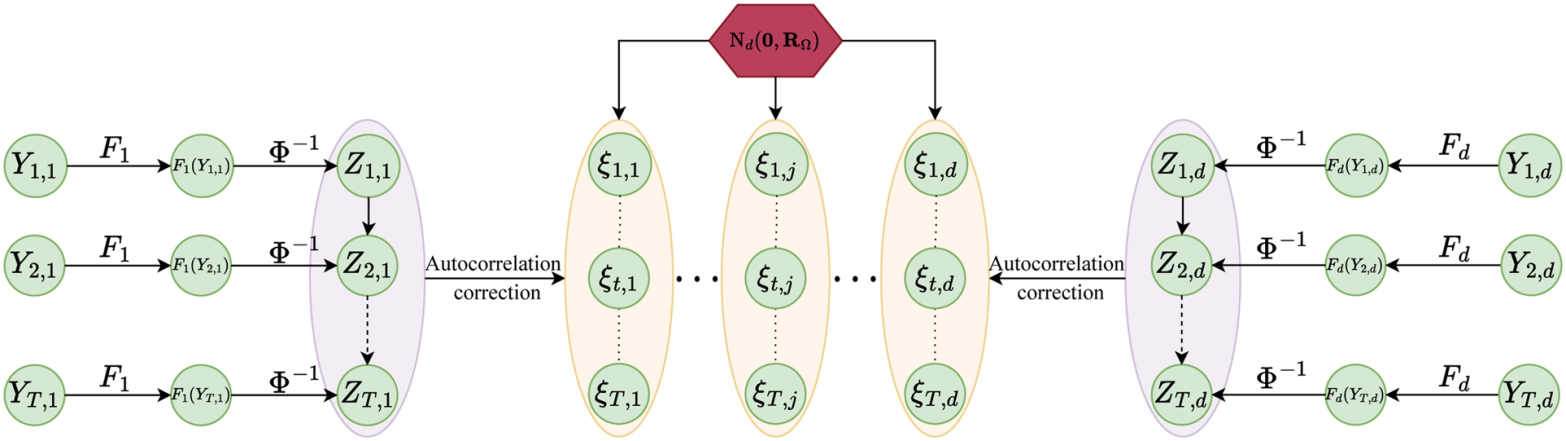
Graphical illustration of the hierarchical structure of the autoregressive matrix-Gaussian copula graphical model (ARMGCGM): For brevity, we omitted the superscript(r,i)—the run and subject indicators, respectively, in this illustration.$Y_{t,j}$is the observed BOLD signal from thej-th ROI at thet-th time point.$Z_{t,j}=\phi^{-1}\left(F_{j}\left(Y_{t,j}\right)\right)$is the BOLD signal in the Gaussian copula space. Probabilistic dependencies exist between$Z_{t,j}$’s across bothjandt; dependence acrossjincurs due to the interaction between the ROIs, whereas dependence acrosstoccurs due to the temporal dependence between successive fMRI scans.$\xi_{t,j}$’s are the autocorrelation-corrected$Z_{t,j}$’s with${\left(\xi_{t,1},\ldots ,\xi_{t,d}\right)}^{\text{T}}\overset{\text{iid}}{∼}\text{N(}\text{0},{\text{R}}_{\text{Ω}}\text{)}$for allt=1,…,T.

We take a Bayesian route to estimation and inference, where we assign priors to the model parameters, and then infer them based on samples drawn from the posterior using a Markov chain Monte Carlo (MCMC) algorithm discussed in detail in theSupplementary Materials. As was shown inChandra et al. (2021), the LRD representation makes the MCMC sampling very efficient via a latent variable augmentation scheme. In theSupplementary Materials, we also discuss a multiple hypothesis testing-based edge discovery procedure that utilizes the posterior uncertainty of the parameters and controls the false discovery rate (FDR) at 10% level in a principled manner.

### 2.4 Priors on the parameters

For the autoregressive parameters inequation (1), for allr,i,jwe assume

$\beta_{1,j}^{\left(r,i\right)},\ldots ,\beta_{L,j}^{\left(r,i\right)\;}|\varsigma_{j}^{2\left(r,i\right)}\overset{iid}{∼}\text{N}\left(0,\nu_{\beta }^{-1}\varsigma_{j}^{2\left(r,i\right)}\right),\;\;\varsigma_{j}^{-2\left(r,i\right)}\overset{iid}{∼}\text{Ga}\left(a_{\varsigma },b_{\varsigma }\right),$where$\nu_{\beta },a_{\varsigma },b_{\varsigma }>0$are fixed hyperparameters, andGa(a,b)denotes a gamma distribution with meana / band variance$a/b^{2}$. For the parameters of the mixture models specifying the marginals inequation (4), we consider the following priors

$$\begin{array}{l} \left({\text{π}}_{1,j}^{\left(r,i\right)},\ldots ,{\text{π}}_{K,j}^{\left(r,i\right)}\right)∼\text{Dir}\left(\frac{\alpha_{\text{π}}}{K},\ldots ,\frac{\alpha_{\text{π}}}{K}\right),\;\;\\ \left(\mu_{h,j}^{\left(r,i\right)},\sigma_{h,j}^{2\left(r,i\right)}\right)\overset{iid}{∼}\text{NIG}\left(\mu_{0},\nu_{0},a_{0},b_{0}\right), \end{array}$$

where$\left(\mu ,\sigma^{2}\right)∼\text{NIG}\left(\mu_{0},\nu_{0},a_{0},b_{0}\right)$is the normal-inverse-gamma prior implying that$\mu |\sigma^{2}∼\text{N}\left(\mu_{0},\nu_{0}^{-1}\sigma^{2}\right)$and$\sigma^{-2}∼\text{Ga}\left(a_{0},b_{0}\right)$.

Ideally,$\left(\beta_{1,j}^{\left(r,i\right)},\ldots ,\beta_{L,j}^{\left(r,i\right)},\varsigma_{j}^{\left(r,i\right)}\right)$should satisfy some constraints to conform with the$Z_{t,j}^{\left(r,i\right)}$’s being stationary and having unit variance. Enforcement of such constraints, however, presents a significant challenge. Instead, in our implementation, we left these parameters unrestricted but observed the posterior to always converge to regions where such properties were accurately satisfied.

Next, we consider a shrinkage prior on the elements ofΛthat shrinks redundant elements ofΛto zero, allowing additional model-based parameter reduction. In particular, we assign a two-parameter generalization of the Dirichlet–Laplace (DL) prior fromBhattacharya et al. (2015)that allows more flexible tail behavior on the elements ofΛ. On ad-dimensional vector$\theta ={\left(\theta_{1},\ldots ,\theta_{d}\right)}^{T}$, our DL prior with parametersaandb, denoted byDL(a,b), can be specified in the following hierarchical manner.

$$\begin{array}{l} \theta_{j}|\varrho ,\phi ,\tau \overset{ind}{∼}\text{N}\left(0,\varrho_{j}\phi_{j}^{2}\tau^{2}\right),\;\;\;\varrho_{j}\overset{iid}{∼}\text{Exp}\left(1/2\right),\;\;\;\\ \phi ∼\text{Dir}\left(a,\ldots ,a\right),\tau \;∼\text{Ga}\left(da,b\right), \end{array}$$

where$\theta_{j}$is thej-th element ofθ,ϕandϱare vectors of same length asθ,Exp(a)is an exponential distribution with mean1/a, and$\text{Dir}\left(a_{1},\ldots ,a_{d}\right)$is ad-dimensional Dirichlet distribution. The original DL prior is a special case withb=1/2. We letvec(Λ)∼DL(a,b).

We use a Dirichlet process (DP) prior (Ferguson, 1973) on the$\delta_{j}^{2}$’s as$\delta_{j}^{2}\left|G\overset{iid}{∼}G,G\right|\alpha ∼\text{DP}\left(\alpha ,G_{0}\right)\text{ with }G_{0}=$$\text{Ga}\left(a_{\delta },b_{\delta }\right),\alpha ∼\text{Ga}\left(a_{\alpha },b_{\alpha }\right),$whereαis the concentration parameter and$G_{0}$is the base measure to favor a smaller number of unique$\delta_{j}^{2}$’s in a model-based manner. The DP model allows clustering the$\delta_{j}^{2}$’s facilitating additional data-adaptive parameter reduction when necessary. Additionally, a DP prior has full prior support on the range space of the number of unique$\delta_{j}^{2}$’s implying a fully flexible model (see Chapter 4 ofGhosal and van der Vaart (2017)).

We discuss a Markov chain Monte Carlo (MCMC)-based strategy of sampling from the posterior of ARMGCGM in Section S1.2 of theSupplementary Materials, where the involving steps are parallelized over the subjects, allowing scalability. In Section S1.3 of theSupplementary Materials, we discuss the choice of hyperparameters used for the analyses presented in this paper. Our implementation using the proposed parallelized MCMC scheme ran in 125 min in a system with 13th Gen Intel(R) Core(TM) i9-13900K CPU and 128 GB RAM, fitting the ARMGCGM to the 12-node auditory network with 7,500 MCMC iterations,*including*both phase-encoding schemes.

## 3 Results

### 3.1 Partial correlations between regions of interest

Using our ARMGCGM approach, we first estimate the precision matrixΩand subsequently compute the partial correlation matrix. We report the significant edges subject to controlling the posterior false discovery rate (Sarkar et al., 2008) at the 10% level. (Details are provided in Section S1.4 in theSupplementary Materials.) InFigure 2we provide the circos plots of the connectivity graphs along with respective weighted adjacency matrices. The(j,h)-th off-diagonal element of the adjacency matrices admit the value 0 if the edge between ROIsjandhis not statistically significant, if the edge is significant then the corresponding partial correlation is plugged in to indicate the strength of the edge.

**Fig. 2.**
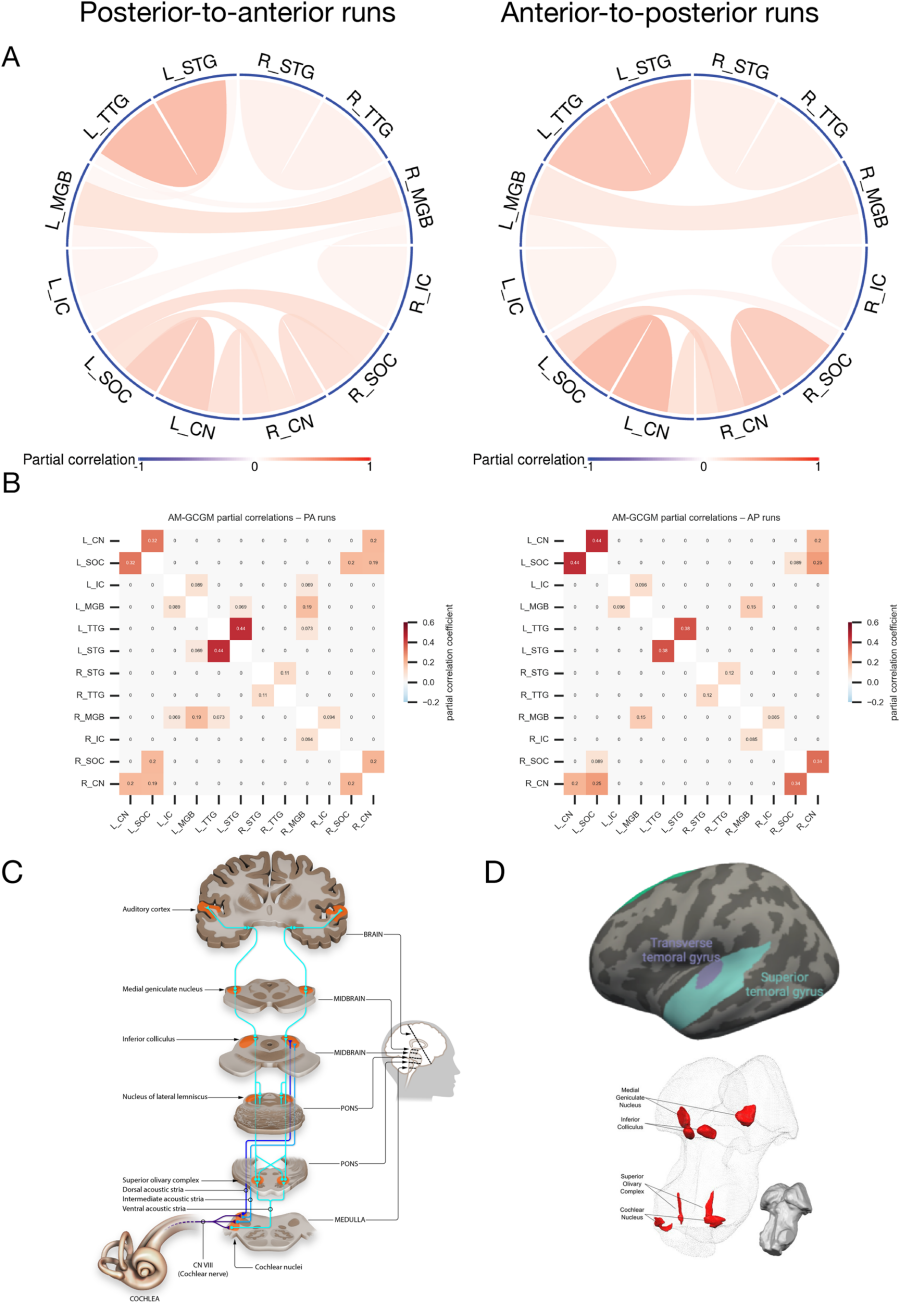
(A) Partial correlation connectivity in data acquired with posterior-to-anterior (PA; left) and anterior-to-posterior (AP; right) phase-encoding directions using the ARMGCGM approach in subcortical and cortical auditory regions. Positive (negative) associations are represented by red (blue) links, their opacities being proportional to the corresponding association strengths. The link widths are inversely proportional to the number of edges associated with the corresponding nodes. (B) The same results as (A), viewed as adjacency matrices (left = PA runs; right = AP runs). (C) Schematic of the auditory pathway from the cochlea through brainstem to cortex (https://osf.io/u2gxc/). (D) Regions of interest from which functional timeseries were extracted. Top: cortical regions from FreeSurfer’s DKT atlas. Bottom: subcortical auditory regions (Sitek et al., 2019).

We found that the strongest auditory connectivity was between adjacent structures in the same hemisphere, particularly between the auditory midbrain (inferior colliculus, or IC) and thalamus (medial geniculate body, or MGB) and between core and associative auditory cortex (TTG and STG). Minimal connectivity was observed between homologous auditory structures across hemispheres. Connectivity was also present between adjacent brainstem auditory structures (cochlear nucleus [CN] and superior olivary complex [SOC]), largely bilaterally. Interestingly, despite the SOC being the primary (and earliest) decussation point in the primary auditory pathway, we only observed partial correlations between right CN and left SOC (in both data partitions), not left CN and right SOC. (SeeSection 4for potential explanations.)

### 3.2 Effect of phase-encoding scheme on subcortical connectivity

Due to the anatomical location of the subcortical auditory structures—in dense, heterogeneous subcortical regions and largely adjacent to CSF—we conducted connectivity analyses separately on AP- and PA-acquired fMRI runs. We then compared the connectivity results from the two acquisition schemes. Overall connectivity patterns were highly similar between the two phase-encoding schemes, as seen in panels A and B ofFigure 2. Subcortical connectivity was quite robust between the brainstem auditory regions. To quantify the similarity between the results in PA and AP acquisitions (plotted inFig. 3A), we computed the Pearson correlation coefficientrbetween the estimated partial correlations. We found a Pearson correlationr=0.940withp-value<0.001between the partial correlations for PA and AP acquisitions that were computed with the proposed ARMGCGM. Note that a large proportion of points overlap at the origin [0.0, 0.0], indicating that ARMGCGM induced sparsity in the connectivity graphs similarly in the two acquisition schemes, which contributes to the high Pearson correlation between the schemes. To assess the similarity in sign of connectivity between acquisition schemes, we computed the Jaccard dissimilarity on the signed off-diagonals of each adjacency matrix. For the proposed ARMGCGM, the Jaccard dissimilarity was 0.231. Additionally, we computed the Euclidean distance between partial correlations in the two acquisition schemes, compared it with some selected approaches from the literature and report the results inTable 1. These results indicate high level of consistency between the findings from the two different acquisition schemes, with the ARMGCGM exhibiting the strongest similarity.

**Fig. 3.**
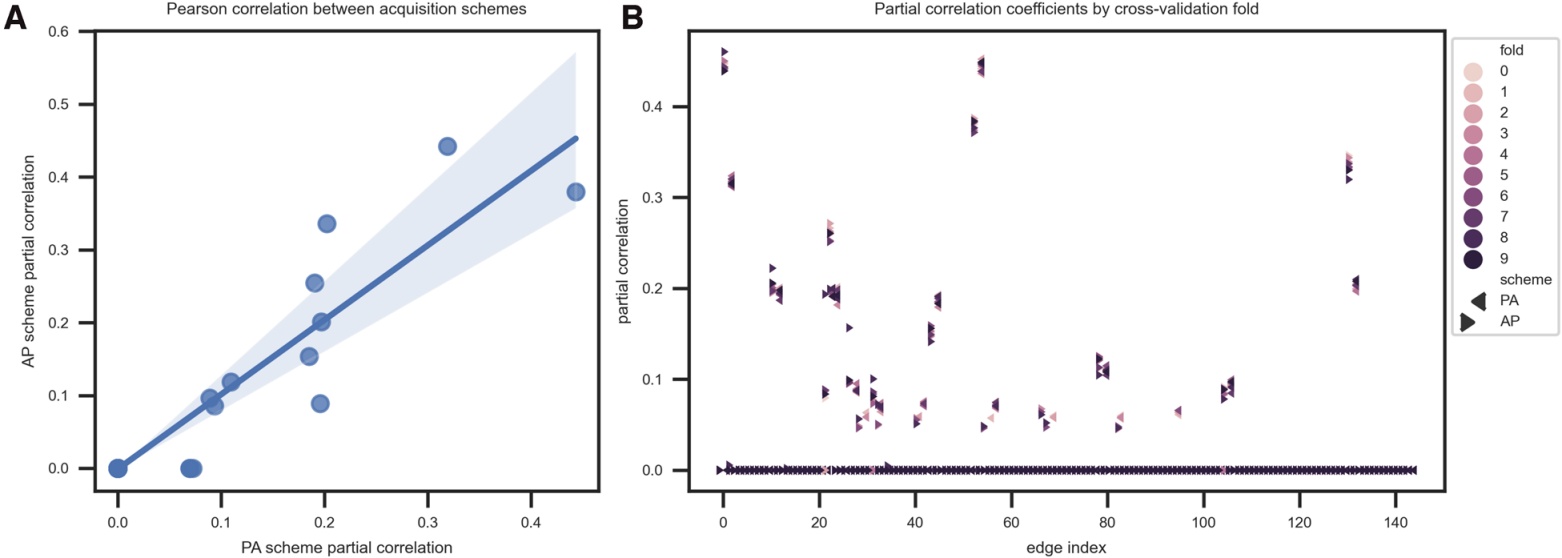
(A) Pearson correlation of partial correlation values between posterior-to-anterior and anterior-to-posterior phase-encoding acquisition schemes across all edges in the graph (region-to-region connections). Note that a high proportion of values overlap at the origin [0.0, 0.0], indicating consistent sparsity across the two acquisition schemes. (B) Partial correlation coefficients (for each region-to-region edge) from the proposed ARMGCGM analysis conducted separately across cross-validation folds (10 folds for each of the two phase-encoding acquisition schemes). Note the large number of edges with partial correlation coefficients of 0.0.

**Table 1.** Several measures of dissimilarities between the functional connectivity networks (using full and partial correlations) estimated in each of the acquisition schemes are reported here.

Method	Pearson’s correlation coefficient	Jaccard dissimilarity	Euclidean distance
ARMGCGM	0.940 ( p -value <0.001 )	0.231	0.262
Full correlation	0.811 ( p -value <0.001 )	0.338	1.016
Glasso	0.749 ( p -value <0.001 )	0.414	0.959
PFM	0.747 ( p -value <0.001 )	0.379	0.975

We provide results for all the correlation-based functional connectivity analyses, viz. ARMGCGM, full correlation, Glasso, and PFA. Note that lower values of Jaccard dissimilarity and Euclidean distance imply better method.

### 3.3 Cross-validation of partial correlations

To assess the stability of our proposed ARMGCGM, we ran leave-10%-out cross-validation by first randomly splitting theNsubjects into 10 equal folds. Then, for each of the 10 folds, we held out the BOLD signals${\left\{Y_{t,j}^{\left(r,i\right)}\right\}}_{t=1}^{T}$for alli’s in that fold and fit ARMGCGM on the remaining 90% subjects. Finally, we compared the estimated partial correlations across the folds. Similar approaches are used in the literature to check the stability of graph estimates (Gan et al., 2018;Mazumder & Hastie, 2012). We repeated this leave-10%-out cross-validation approach for both phase-encoding schemes. Partial correlation coefficients for each of the 20 folds (10 folds for each of the 2 acquisition schemes) are presented inFigure 3B, which shows highly similar partial correlation estimates across all folds and both phase-encoding schemes (intraclass correlation of edgewise partial correlations across cross-validation folds = 0.991).

In Section S1.7 of theSupplementary Materials, we performed additional simulation studies (Supplementary Fig. S5) which illustrates our method’s ability to efficiently recover the “true” underlying connectivity graph.

### 3.4 Comparison with existing approaches

We compared with three standard approaches in the literature: (1) correlation analysis between the ROIs (Biswal et al., 1995;Cordes et al., 2000;Fox & Raichle, 2007;Lowe et al., 1998;Smith, Vidaurre, et al., 2013); and partial correlation analyses using (2) the graphical lasso (Friedman et al., 2008) and (3) the precision factor model (Chandra et al., 2021). In all comparisons, we did separate analyses for each of the acquisition schemes.

#### 3.4.1 Comparison 1: full correlation approach

Here we study the marginal correlation between the ROIs. Letting$\rho_{j,j^{\prime }}$denote the correlation between ROIsjand$j^{\prime }$in resting state we test

$$H_{0,j,j'}:|\rho_{j,j'}|=0\;\text{versus}\;H_{1,j,j'}:|\rho_{j,j'}|\neq 0\;for\;all\;1\leq j<j'\leq d.$$.

For each acquisition scheme, we first concatenate the timeseries across all runs and individuals. Then we performt-tests for correlation for all$\left(j,j^{\prime }\right)$pairs followed by the Benjamini–Hochberg false discovery rate (FDR) correction (Benjamini & Hochberg, 1995) for multiplicity adjustment and control the FDR at level0.10. Traditionally, full correlations are used to measure functional “connectivity” in resting-state fMRI studies (Biswal et al., 1995;Cordes et al., 2000;Lowe et al., 1998). InFigure 4, we provide the correlation graphs and correlation matrices separately for each acquisition scheme. We find the correlation graphs to be much denser compared with the partial correlation graphs presented inFigure 2. Unlike in the ARMGCGM method, we observed negative values when using full correlations (particularly in the PA acquisition scheme), although these negative correlations are generally close to 0. In this full correlation approach, the similarity between runs split by data acquisition scheme was characterized by Pearson’srof0.811(p-value<0.001) and a Euclidean distance of 1.016. The Jaccard dissimilarity of the signed adjacency matrix was 0.338.

**Fig. 4.**
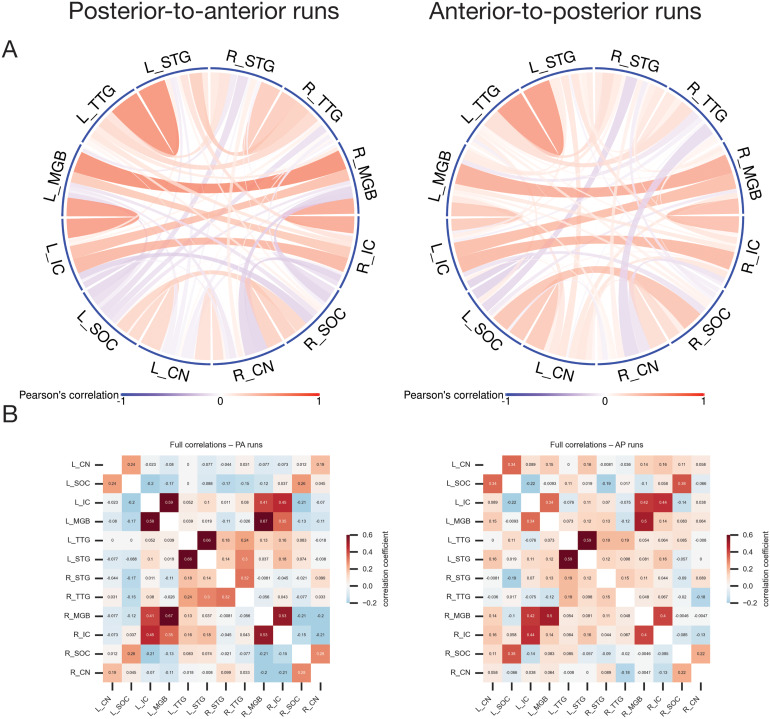
(A) Full correlation connectivity in data acquired with posterior-to-anterior (PA; left) and anterior-to-posterior (AP; right) phase-encoding directions using*t*-tests. Positive (negative) associations are represented by red (blue) links, their opacities being proportional to the corresponding association strengths. The link widths are inversely proportional to the number of edges associated with the corresponding nodes. (B) The same results as (A), viewed as adjacency matrices (left = PA runs; right = AP runs).

#### 3.4.2 Comparison 2: partial correlations with Glasso approach

We first consider the graphical lasso (Glasso) approach (Friedman et al., 2008) as another alternative choice for partial correlation-based conditional graph estimation. Glasso assumes iid data from a multivariate Gaussian distribution (i.e., without any correction for autocorrelation) with l_1__( ⋅ )_penalty on the precision matrix. In this analysis, we concatenated the$Y_{t,j}^{\left(r,i\right)}$values across(r,i)and created a(TR)×dmatrix, sayY, for each acquisition scheme, and applied the Glasso model onY. We use 10-fold cross-validation to choose the optimal penalty parameter. The Glasso approach provides a point estimate of the sparse precision matrix and hence the functional connectivity network. In panels A and B ofFigure 5, we plot the connectivity graphs and weighted adjacency matrices, respectively, separately for each acquisition scheme.

**Fig. 5.**
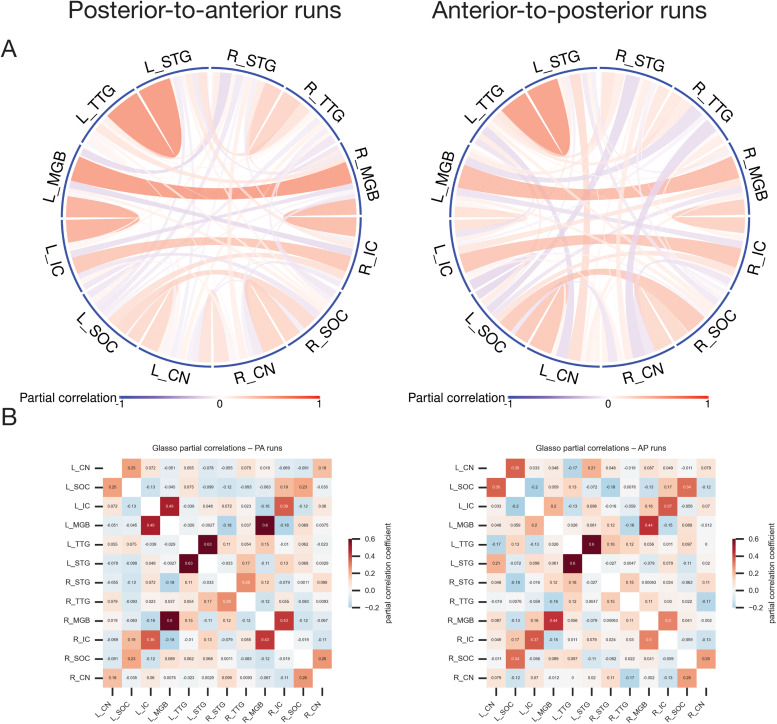
(A) Partial correlation connectivity in data acquired with posterior-to-anterior (PA; left) and anterior-to-posterior (AP; right) phase-encoding directions using the Glasso approach. Positive (negative) associations are represented by red (blue) links, their opacities being proportional to the corresponding association strengths. The link widths are inversely proportional to the number of edges associated with the corresponding nodes. (B) The same results as (A), viewed as adjacency matrices (left = PA runs; right = AP runs).

These functional connectivity graphs inFigure 5are much denser compared with the estimates obtained by our proposed ARMGCGM inFigure 2. To quantify the robustness and stability of the Glasso graphs in this application, we compute Pearson correlation coefficient and Euclidean distance between the estimated partial correlations, and the Jaccard dissimilarity between the signs of the adjacency matrices in PA and AP acquisitions in the same manner as we did for ARMGCGM elaborated inSection 3.2. We reported the values inTable 1.Figure 5indicates that the strong positive correlations are consistent across the acquisition schemes. However, substantial discrepancy can be observed for the weak edges, particularly for the negative partial correlations. This is a common phenomenon for graphical models if the Gaussian assumption is made on non-Gaussian distributed data; see, e.g.,Section 5, example 1(a) inGuha et al. (2020).

#### 3.4.3 Comparison 3: partial correlations with PFM

In this analysis, we concatenated the$Y_{t,j}^{\left(r,i\right)}$values across(r,i)and created a(TR)×dmatrix, sayY, for each acquisition scheme, and applied the PFM onY. Like Glasso, the PFM also assumes iid data from a multivariate Gaussian distribution and does not correct for temporal autocorrelations in the data. We infer on the graph using the Bayesian multiple comparison technique described in theSupplementary Materials. Results are provided inFigure 6. The connectivity graphs majorly differed with our ARMGCGM results, with the PFM-derived graph being much denser and includes more (weakly) negative edges. In the top panel ofFigure 7, we plot the marginal Gaussian fits on the histograms of some BOLD signals. Clearly the simple Gaussian assumption does not hold here and a more sophisticated approach like ours is required.

**Fig. 6.**
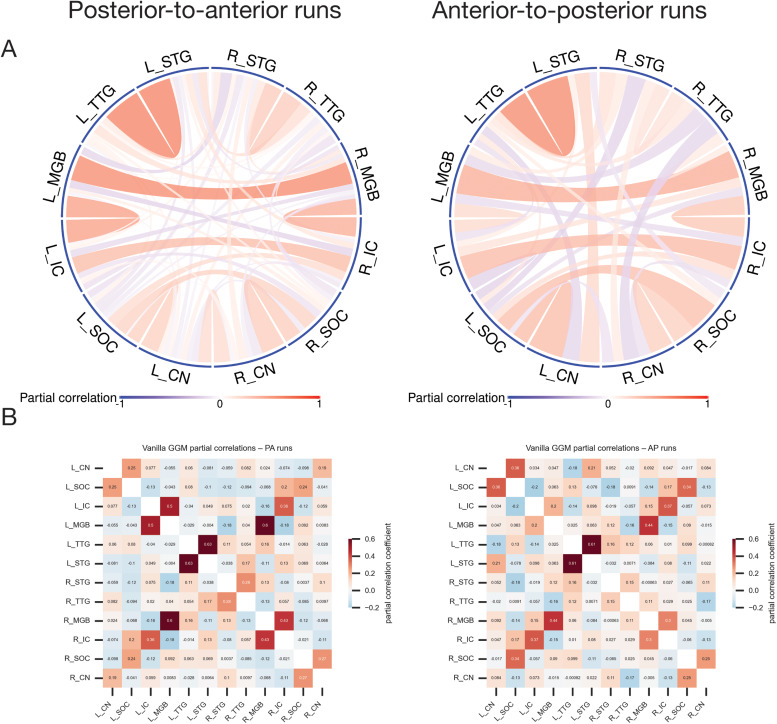
(A) Partial correlation connectivity in data acquired with posterior-to-anterior (PA; left) and anterior-to-posterior (AP; right) phase-encoding directions using the PFM (comparison 3). Positive (negative) associations are represented by red (blue) links, their opacities being proportional to the corresponding association strengths. The link widths are inversely proportional to the number of edges associated with the corresponding nodes. (B) The same results as (A), viewed as adjacency matrices (left = PA runs; right = AP runs).

**Fig. 7.**
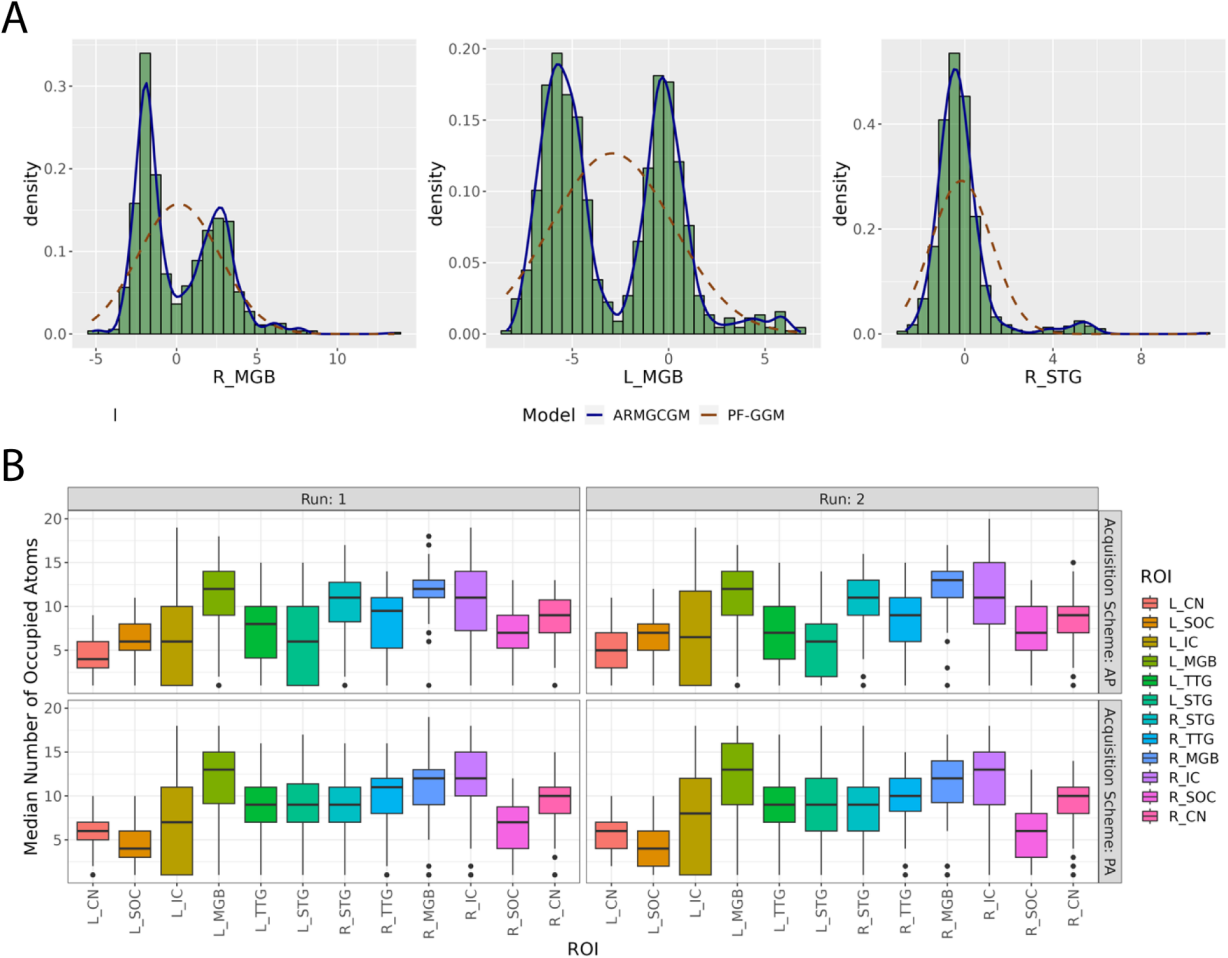
Marginal fits for the location-scale mixture of Gaussian distributions. (A) The sample histograms in green show that distribution of the ROIs substantially deviates from Gaussian assumption. The blue lines are the respective fitted densities corresponding to the mixture model inequation (4). These figures indicate excellent goodness-of-fit. The dashed brown lines correspond to marginal Gaussian fits of the vanilla PFM which were evidently underfitted. (B) The boxplots of the number of occupied clusters across subjects corresponding to the mixture model indicate that our model specifications are adequate.

To assess the robustness and stability of the PFM, we computed Pearson correlation coefficient and Euclidean distance between the estimated partial correlations, and the Jaccard dissimilarity between the signs of the adjacency matrices in PA and AP acquisitions in the same manner as we did for ARMGCGM elaborated inSection 3.2. We reported the values inTable 1, where we see that PFM exhibits more consistency than Glasso but ARMGCGM performed best.

### 3.5 Fit of the autoregressive matrix-Gaussian copula graphical model

#### 3.5.1 Density fits

We studied the goodness-of-fit of the proposed ARMGCGM. In the top panel ofFigure 7, we plotted the sample histograms and the corresponding fitted marginal densities for some selected ROIs. The sample histograms strongly indicate the marginal distributions of the data to deviate substantially from Gaussian distributions, including some with multiple well-separated modes.Figure 7also shows that our flexible location-scale mixture of Gaussians fits the data very well, even for the most complicated distributions.

Mixtures of Gaussians with reasonably large number of mixture components can approximate large classes of widely varying distributions. To validate whether the number of mixtures(K=20)in model (4) is adequate, we computed the median number of nonempty clusters across MCMC samples for each subject and ROI in each run. In the bottom panel ofFigure 7, we plotted the histograms of the medians across the subjects. As the number of nonempty clusters is smaller thanKconsistently across all setups yielding excellent fits for complicated distributions, we conclude that our model specifications are adequate.

#### 3.5.2 Autocorrelation corrections

We recall fromSection 2.3that$Z_{t,j}^{\left(r,i\right)}$’s were the transformed BOLD signal timeseries in the Gaussian space and$\xi_{t,j}^{\left(r,i\right)}$’s were the autocorrelation-corrected timeseries. To check for autocorrelation corrections using model (1), we plotted the partial autocorrelations between$Z_{t,j}^{\left(r,i\right)}$’s and$\xi_{t,j}^{\left(r,i\right)}$’s across time for all ROIs. Since theZandξvalues vary across MCMC iterations, we used their posterior expectations in this analysis. InFigure 8, we plot the partial autocorrelations for a randomly selected individual in run 1 of posterior-to-anterior acquisition scheme; the blue horizontal dashed lines represent the 5% band. The figure clearly indicates that the autoregressive model of orderL=5corrects for the autocorrelations. As we obtained very similar results for other runs and acquisition schemes, we have omitted them from the paper.

**Fig. 8.**
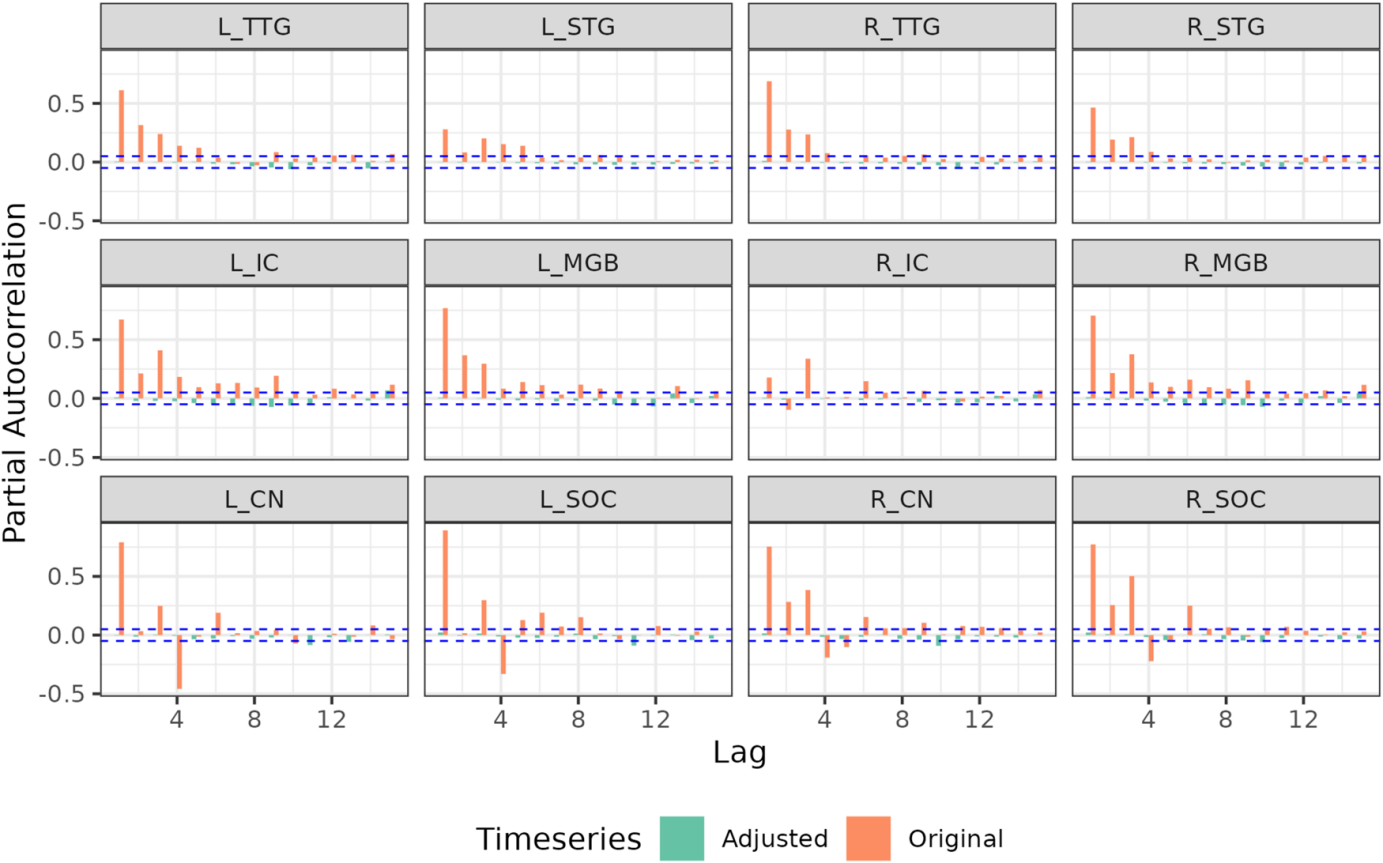
Partial autocorrelation plots of the fMRI timeseries for a randomly selected subject in the posterior-to-anterior acquisition scheme across the ROIs before (orange) and after (green) autocorrelation corrections; the horizontal dashed blue lines represent the 5% band. The plot indicates that autocorrelations are corrected in our model.

## 4 Discussion

The mammalian auditory pathway consists of a series of obligatory and interconnected subcortical and cortical brain structures. Assessing the connectivity of the human subcortical auditory structures has been limited due to methodological challenges of noninvasive imaging of the deep, small structures. Recent acquisition and analytical advances enable finer grained investigations of connectivity throughout the brain, including the brainstem. In this paper, we validated a novel autoregressive matrix-Gaussian copula graphical model to estimate functional auditory connectivity patterns from a publicly available high-resolution resting-state functional MRI dataset. Using partial correlations (as opposed to full correlations) allowed us to identify specific relationships between nodes in a connectivity graph by removing shared variance across nodes (SupplementaryFigs. S1 and S2). We found highly consistent connectivity patterns between adjacent auditory brain regions along the auditory pathway that demonstrate the efficacy of our connectivity method as well as the potential for functional connectivity investigations of the subcortical auditory system. Below, we separately discuss our novel scientific findings and our novel contributions to the statistics literature.

### 4.1 Novel contributions to the human auditory neuroscience literature

To date, there have been only limited applications of functional MRI methods to study subcortical auditory connectivity (Berlot et al., 2020;Hofmeier et al., 2018). Using our novel ARMGCGM approach in the present study, we found strong partial correlations between cochlear nucleus (CN) and superior olivary complex (SOC) bilaterally using resting-state functional MRI data. Most interestingly, we observed contralateral CN–SOC connectivity between right CN and left SOC (and in both data acquisition schemes), fitting the ground truth primary auditory pathway crossing from left to right (and vice versa) between CN and SOC (Barnes et al., 1943;Schofield, 1994). These functional connectivity patterns between CN and SOC have not been previously observed in human auditory brainstem in vivo but follow our understanding of the mammalian primary auditory pathway based on research in animal models (Barnes et al., 1943;Doucet & Ryugo, 2003;Harrison & Irving, 1966). Principally, auditory information that is transduced by the cochlea of each ear is transmitted via the cochlear nerve to the cochlear nucleus, the first stage of the central auditory pathway, on each side of the brainstem. In the primary auditory pathway, the lemniscal anteroventral subdivision of the cochlear nucleus enhances the fine temporal precision of incoming auditory signals (Pickles, 2015). From there, auditory signals are passed to both the ipsilateral and contralateral SOC for further auditory processing, including spatial localization (Moore, 2000). The SOC comprises multiple distinct subdivisions, which receive ipsilateral and contralateral connections from cochlear nucleus to varying degrees (Pickles, 2015), aligning with our overall bilateral connectivity results between CN and SOC.

While we observed consistent right CN and left SOC connectivity, it is unclear why similar patterns were not observed between left CN and right SOC. One contributor is the lower signal-to-noise ratio in fMRI data from the lower brainstem. Paired with the small size of each of the brainstem auditory nuclei, we may still be at the edge of what is detectable using present functional connectivity methods. Additionally, this analysis was conducted on “resting-state” fMRI data, during which no auditory stimuli of interest was presented or overt tasks were conducted. Resting-state fMRI connectivity in the cochlear nucleus and superior olivary complex has not been examined in the previous literature to our knowledge; it is possible that sound-evoked connectivity methods would evoke greater functional connectivity, particularly in these earliest stages of the auditory pathway. Further, ipsilateral connections (i.e., between left CN and left SOC and between right CN and right SOC) may be artifactually stronger due to their physical proximity. Even with relatively high 1.05 mm spatial resolution 7T fMRI data, CN and SOC on each side are only separated by a few voxels. These regions are thus at increased likelihood of sharing temporal fluctuations due to partial volume effects, wide point-spread functions, spatial dependence, or another as-yet-unsolved fMRI confounds that are particularly acute in the lower brainstem.

Moving up the primary auditory pathway, we observed significant partial correlations between ipsilateral inferior colliculus (IC) in midbrain and medial geniculate body (MGB) of the thalamus in both hemispheres and in both phase-encoding schemes. Inferior colliculus is a major convergence point in the auditory system, with the lemniscal IC subdivision being thought to convert distinct auditory features into discrete auditory objects for the first time in the auditory pathway. MGB continues the refinement of auditory objects via direct lemniscal connections from IC as well as rich corticofugal connections from auditory cortex to nonlemniscal MGB subdivisions (Pickles, 2015). Interestingly, we found strong partial correlations between left and right MGB in both datasets. Although not directly connected by large white matter bundles, left and right MGB are expected to process auditory information from IC at similar levels of abstraction. Thus, partial correlations may reflect indirect but shared neural mechanisms of auditory processing in the thalamus.

We did not observe IC partial correlation connectivity with either brainstem or cortical structures. IC is a key hub in the auditory system, receiving bottom-up sensory information as well as top-down modulating signals from auditory cortex and other brain regions. The lack of partial correlation connectivity with IC may be due to strong IC subdivision-specific functionality, with IC core primarily serving an ascending lemniscal role and dorsal and external IC having top-down and nonlemniscal functions. Averaging over these subdivisions may obfuscate specific connectivity patterns. Alternatively, our results may suggest that it does not have a specialized relationship with any one region beyond MGB but rather integrates and transforms auditory and other neural signals.

Finally in auditory cortex, transverse temporal gyrus (TTG)—the location of primary auditory cortex—was strongly connected with ipsilateral superior temporal gyrus (STG), which contains secondary and associative auditory cortices. Primary auditory cortex receives direct input from lemniscal MGB and is the last auditory structure with fine-grained tonotopicity (Pickles, 2015). In humans, STG is hierarchically structured, with portions further away from primary auditory cortex having increasingly wider temporal integration windows (Hamilton et al., 2018;Norman-Haignere et al., 2022) and greater categorical specificity (Bhaya-Grossman & Chang, 2022;Feng et al., 2021;Hamilton et al., 2020;Keshishian et al., 2023;Nourski et al., 2018;Pernet et al., 2015;Rauschecker & Tian, 2000;Rupp et al., 2022). While invasive recordings from human STG suggest a potential direct connection between MGB and posterior STG (Hamilton et al., 2021), we found mixed evidence for such a direct pathway in our partial correlation data (in one hemisphere in only one of the data partitions). Our partial correlation functional connectivity results align with a vast literature demonstrating information flow between primary and nonprimary auditory cortex (for review, seeHackett (2011). The lack of contralateral partial correlation approach measures the correlation between the concerned ROIs after filtering out the indirect effects of the remaining ROIs. Complementarily, some literature suggest that left and right auditory cortices process auditory information at distinct timescales and levels of abstraction (Güntürkün et al., 2020;Hickok & Poeppel, 2007;Zatorre et al., 2002), with the left auditory cortex being uniquely tuned to rapidly changing temporal information—such as the phonetics of speech sounds—while the right auditory cortex is more sensitive to slower changes in the spectral domain, particularly for speech prosody as well as music.

In comparison with our ARMGCGM partial correlation approach, we computed full correlations in the same network. Connections were much denser in the full correlation approach, aligning with the rich interconnectedness of the auditory system (Pickles, 2015). Unlike with partial correlations, which highlighted hierarchical connections between adjacent nodes along the auditory pathway, we observed positive full correlations between all auditory cortical regions, regardless of hemisphere. Additionally, we found a strong positive subnetwork including IC and MGB bilaterally, whereas many of these connections (such as between left and right IC) were absent in the ARMGCGM partial correlation analysis. Since partial correlations characterize connectivity between two nodes after filtering out the effects of the other nodes, our combined results point to widespread shared information across the auditory system (per full correlation analysis) with additional shared processing between adjacent nodes of the canonical auditory pathway (per partial correlation analysis). This suggests distinct but complementary use of full and partial correlations, with full correlation analysis identifying a rich network of interconnected nodes, while partial correlations are sensitive to strong node-to-node connections.

### 4.2 Novel contributions to the graphical model literature

In this article, we developed an autoregressive matrix-Gaussian copula graphical model (ARMGCGM) for non-Gaussian distributed data with temporal autocorrelation, the problem of estimating brain connectivity patterns from resting-state fMRI data being the primary motivation. In the ARMGCGM, we begin by employing flexible individual-specific and brain-region-specific location-scale mixtures of Gaussians to model the marginal distributions of the observed fMRI data. Subsequently, these models are utilized to transform the observed data into latent Gaussian timeseries. We then apply higher-order autoregressive models to capture the temporal dependence in this transformed space within each region. Finally, a Gaussian copula is employed on the autocorrected timeseries to capture the conditional dependencies between different brain regions shared across individuals. The ARMGCGM thus allows borrowing of information across different subjects to infer on a common connectivity graph while taking into account subject and run-specific variability via flexible mixture models. We leverage recent advances on modeling precision matrices via a flexible but computationally efficient low-rank-diagonal decomposition method that not allows efficient exploration of the posterior space for estimating the connectivity graph. Compared with alternative approaches to exploiting partial correlations to estimate connectivity graphs, our proposed ARMGCGM method produces results that are more consistent across fMRI data acquisition schemes with respect to multiple metrics. Additionally, the results remain highly stable in a leave-10%-out cross-validation. Considering the low signal-to-noise ratio in BOLD signals from deep small auditory structures, our results demonstrate the sensitivity and specificity of our model to neurobiologically plausible connections. While the proposed ARMGCGM offers a more sophisticated method for modeling fMRI data, with the potential to fit arbitrarily complex marginal distributions, it comes with a higher numerical cost compared with simplistic parametric models. However, our parallelized Markov chain Monte Carlo implementation runs across all participants in just over 2 hours, showcasing a manageable computation time considering the dataset’s scale. In our view, the enhanced inference capabilities outweigh the computational burden.

### 4.3 Comparisons with connectivity literature

Our study is the first to systematically assess connectivity across the human auditory pathway using multiple connectivity measures, with previous subcortical connectivity studies limited to full correlation analysis (Berlot et al., 2020;Hofmeier et al., 2018;Leaver et al., 2016;J. Zhang et al., 2015). Given the anatomical and methodological constraints with subcortical fMRI, the limited fMRI connectivity literature is not too surprising. First, the deep location of the subcortical structures places them far from MRI transmit and receive coils, limiting the signal-to-noise ratio from these regions (Miletić et al., 2020). Because the brainstem is relatively centrally located relative to the multiple receiver coils, accelerated acquisition techniques that are based on phase differences between receiver coils are less effective (Preibisch et al., 2015). Second, subcortical nuclei can be quite small, requiring higher resolution imaging protocols (which unfortunately trade off SNR in order to achieve greater spatial resolution). Third, subcortical nuclei are densely organized adjacent to nuclei with heterogeneous functions, so voxels immediately next to those containing core auditory structures could contain visual, motor, or sensory nuclei, white matter, CSF, or a combination of any of these. Ultimately, each of these constraints limits the SNR from subcortical auditory nuclei.

Constraints in human subcortical auditory research have translational consequences beyond basic science. For instance, while cochlear implants have been widely successful at providing sensory information to individuals with sensorineural hearing impairments with an intact cochlear nerve (Kral et al., 2019;Reiss, 2020), auditory prostheses in the central auditory system have been less successful (Lim et al., 2009;Shetty et al., 2021), due in no small part to our limited understanding of the complexity of sound representation in the ascending auditory pathway.

### 4.4 Limitations and future directions

As the canonical neuroanatomy of the primary auditory pathway is consistent across individuals and well described in the literature (Sitek et al., 2019), we have the*a priori*expectation of a shared auditory graph across all participants. As the goal of this study was to map a network that is strongly expected based on anatomy and nonhuman neurophysiology, we built a joint model that includes data from all participants. This is similar to approaches used in group independent component analysis (Calhoun et al., 2001) and cohort-level brain mapping (Varoquaux et al., 2013). However, subject-specific differences in the distributions of the BOLD signals as well as autocorrelations between successive scans can induce artifactual and noisy edges in functional connectivity graphs, as seen in the Glasso and PFMs; we take care of these issues in ARMGCGM. Nevertheless, we did not investigate differences in functional connectivity between participants in the current article. Building on ARMGCGM to explore how functional connectivity varies between individuals and groups or as a function of behavior is a priority for future work.

In general, resting-state fMRI connectivity measures become more reliable with longer scans (Zuo et al., 2019). Measurement correlations increase as time in the scanner increases, from Pearson’sr=0.82at 9 min tor=0.92at 27 min tor=0.97at 90 min (Laumann et al., 2015). Others described improved intraclass correlation coefficients with datasets beyond 20 minutes and up to 50 minutes (Xu et al., 2016). The Midnight Scan Club group (Gordon, Laumann, Gilmore, et al., 2017) computed a range of network connectivity metrics and found that reliability generally required at least 30 minutes of resting-state data per subject. One paper (Greene et al., 2020) specifically investigated functional connectivity in subcortical structures and found even longer scan requirements (up to 100 minutes) for subcortical structures due to decreased signal-to-noise ratios deeper in the brain. Additionally, primary sensory networks are among the most stable within and across participants (Gratton et al., 2018;Hutchison et al., 2013). We, therefore, believe it is appropriate and necessary to use datasets with longer scans of resting-state data in order to investigate even static subcortical auditory connectivity.

Further, while many brain networks exhibit temporal dynamics that can tell us about mental state (Fornito et al., 2012) or disease ((Sakoğlu et al., 2010); seeHutchison et al. (2013)for a review), functional connectivity within primary sensory networks is among the most stable over time (Gratton et al., 2018), as they share bidirectional physical connections, share contributions to the same physiological tasks, and are evolutionarily conserved across species (Hutchison & Everling, 2012). In the present work, we were interested in characterizing the stationary connectivity in the primary auditory pathway that is present at rest across individuals. Adapting time-varying dynamics into this model is a promising future direction, particularly if we are interested in higher level cognitive brain networks that vary as a function of task or mental state. Additionally, exploring recent advancements in the statistics literature (Ni et al., 2020;Song et al., 2020) to scale up MCMC implementations of Bayesian mixture models, exploring rank (Hoff, 2007), and empirical likelihood (Mengersen et al., 2013)-based methods for marginal estimation, etc. is of future interest. Finally, adapting our proposed undirected connectivity methods to estimate*directed*graphs holds promising neurobiological value and is a priority for ongoing and future research directions.

## 5 Conclusions

In this article, we validated a novel autoregressive matrix-Gaussian copula graphical model for partial correlation estimation while appropriately correcting for temporal autocorrelations. Using this approach, we identified functional connectivity in the human auditory system using resting-state functional MRI. Whereas a complementary approach using full correlations identified a rich network of interconnected auditory regions, partial correlations highlighted direct connections between adjacent structures along auditory pathways. In particular, subcortical connectivity was highly consistent across acquisitions, demonstrating the utility and applicability of functional connectivity methods in deep brain structures. In the future, we plan to investigate whole-brain partial correlation connectivity across sensory, motor, and higher cognitive networks using the proposed models and their relationship with behavior across individuals.

## Supplementary Material

Supplementary Material

## Data Availability

Data are publicly available through the Human Connectome Project (https://www.humanconnectome.org/study/hcp-young-adult). Analysis code is available in the Supplementary Materials.
