# Supplementary material for: Functional connectivity across the human subcortical auditory system using an autoregressive matrix-Gaussian copula graphical model approach with partial correlations
Source: Imaging Neurosci (Camb). 2024 Aug 12;2:imag-2-00258. doi: 10.1162/imag_a_00258 (PMC11485223; doi:10.1162/imag_a_00258)
Supplement: Supplementary Material [file imag_a_00258-supp.pdf]

S1. Supplementary Materials

S1.1 Full vs Partial Correlation

Here, we illustrate the difference between full and partial correlation analyses in a stylized example. We consider the following full correlation matrix between regions of interest (ROIs) in the left panel of Supplementary Figure S.1 and on the right panel on the same figure, we show the marginal dependency graph. We can see that marginally all of the nodes are dependent on each other.

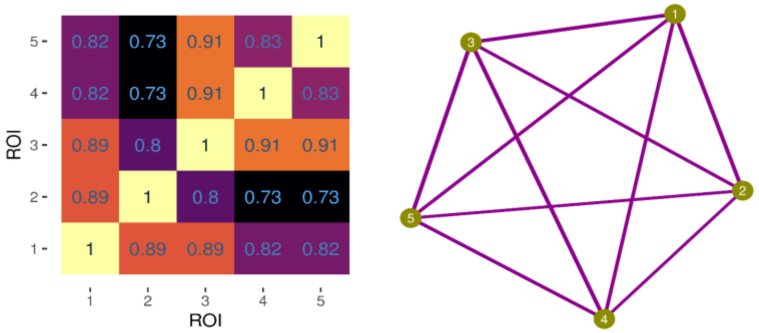

Figure S.1. Simple correlation matrix (left) and marginal dependency graph (right).

We calculate the partial correlation matrix and show in the left panel of Supplementary Figure S.2. Partial correlations represent the dependence between two variables after removing the effects of the other variables. For example, 0.58 is the partial correlation between ROI-5 and ROI-3 after removing the effects of ROIs 1, 2 and 4. On the left panel of Figure S.2 we show the corresponding conditional dependency graph. Even though the marginal graph is dense, we see that the conditional dependence graph is very sparse.

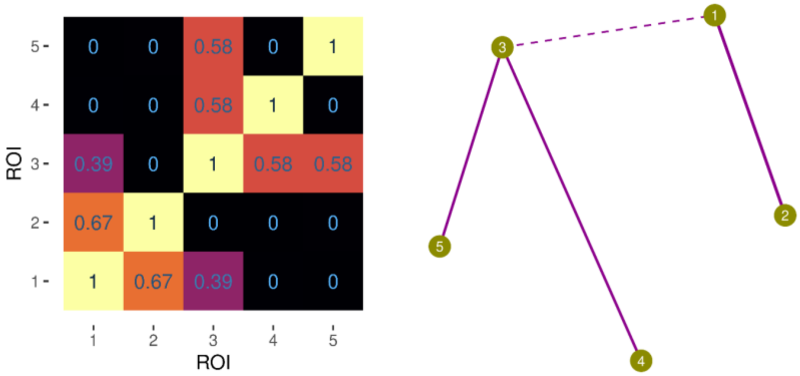

Figure S.2. Partial correlation matrix (left) and conditional dependency graph (right).

S1.2 Posterior Computation

For each ROI, we have timeseries of length T from each of the n subjects. Our Gibbs sampler algorithm to draw samples from the posterior distribution cycles through the following steps.

Step 1 Update the mixture model parameters: Note that the mixture model in equation (4) of the main paper can be equivalently represented as

$$Y_{t,j}^{(r,i)} \mid c_{t,j}^{(r,i)} = h \sim \mathrm{N}\left(\mu_{h,j}^{(r,i)}, \sigma_{h,j}^{2(r,i)}\right), \quad \Pr\left(c_{t,j}^{(r,i)} = h\right) = \pi_{h,j}^{(r,i)} \text{ for all } t = 1, \dots, T, \tag{1}$$

where $c_{t,j}^{(r,i)}$s are latent cluster membership indicators. We update these latent variables and the parameters in the following steps.

Parallel for $i = 1, \dots, N$

for $j = 1, \dots, d$ and $r = 1, \dots, R$

i) for $t = 1, \dots, T$ and $h = 1, \dots, K$, update $c_{t,j}^{(r,i)}$ by sampling $\Pr\left(c_{t,j}^{(r,i)} = h\right) \propto \pi_{h,j}^{(r,i)} \mathrm{N}\left(\mu_{h,j}^{(r,i)}, \sigma_{h,j}^{2(r,i)}\right)$;

ii) for $h = 1, \dots, K$ define the set $S_{h,j}^{(r,i)} = \left\{t: c_{t,j}^{(r,i)} = h\right\}$ and the quantities $N_{h,j}^{(r,i)}$ = cardinality of $S_{h,j}^{(r,i)}$, $\hat{\nu} = \nu_0 + N_{h,j}^{(r,i)}$, $\hat{a} = a_0 + \frac{N_{h,j}^{(r,i)}}{2}$, $\overline{Y}_{h,j}^{(r,i)} = \frac{1}{N_{h,j}^{(r,i)}} \sum_{t \in S_{h,j}^{(r,i)}} Y_{t,j}^{(r,i)}$, $\hat{\mu} = \frac{\nu_0 \mu_0 + N_{h,j}^{(r,i)} \overline{Y}_{h,j}^{(r,i)}}{\hat{\nu}}$, $\hat{b} = b_0 + \frac{1}{2} \sum_{t \in S_{h,j}^{(r,i)}} \left(Y_{t,j}^{(r,i)} - \overline{Y}_{h,j}^{(r,i)}\right)^2 + \frac{N_{h,j}^{(r,i)} \nu_0}{2\hat{\nu}} \left(\overline{Y}_{h,j}^{(r,i)} - \mu_0\right)^2$. Then sample $\left(\mu_{h,j}^{(r,i)}, \sigma_{h,j}^{2(r,i)}\right) \sim \mathrm{NIG}(\hat{\mu}, \hat{\nu}, \hat{a}, \hat{b})$;

iii) sample $\left(\pi_{1,j}^{(r,i)}, \dots, \pi_{K,j}^{(r,i)}\right) \sim \mathrm{Dir}\left(N_{1,j}^{(r,i)} + \frac{\alpha_\pi}{K}, \dots, N_{K,j}^{(r,i)} + \frac{\alpha_\pi}{K}\right)$.

Step 2 Update the autoregressive process parameters: This cycles through the following steps

Parallel for $i = 1, \dots, N$

for $j = 1, \dots, d$ and $r = 1, \dots, R$

(i) Calculate $Z_{t,j}^{(r,i)} = \Phi^{-1}\left\{F_j^{(r,i)}\left(Y_{t,j}^{(r,i)}\right)\right\}$ where $\Phi(\cdot)$ is the CDF of a standard Gaussian distribution and $F_j^{(r,i)}(\cdot)$ is the CDF induced by the mixture model (*1*).

(ii) Letting $\boldsymbol{Z}^{(r,i)} = \left(\left(Z_{t,j}^{(r,i)}\right)\right)_{T \times d}$ denote the matrix of fMRI signals corresponding to the i-th individual in the r-th run in the transformed Gaussian space, obtain $\widetilde{\boldsymbol{Z}}^{(r,i)} = \boldsymbol{Z}^{(r,i)} \boldsymbol{R}_{\Omega}^{-\frac{1}{2}}$.

(iii) Letting $\widetilde{\boldsymbol{Z}}_j^{(r,i)} = \left(\tilde{Z}_{j,1}^{(r,i)}, \tilde{Z}_{j,2}^{(r,i)}, \dots, \tilde{Z}_{j,T}^{(r,i)}\right)^T$ denote the j-th column of $\widetilde{\boldsymbol{Z}}^{(r,i)}$, we define the $T-1$ component vector $\boldsymbol{\eta}_j^{(r,i)} = \left(\tilde{Z}_{j,2}^{(r,i)}, \dots, \tilde{Z}_{j,T}^{(r,i)}\right)^T$ and the $(T-1) \times L$ matrix $\boldsymbol{X}_j^{(r,i)} = ((\tilde{Z}_{j,t-k}^{(r,i)}))_{t=2:T,k=1:L}$ with the convention $\tilde{Z}_{j,t'}^{(r,i)} = 0$ for any $t' \le 0$. Then sample

$$\boldsymbol{\beta}_j^{(r,i)} = \left(\beta_{1,j}^{(r,i)}, \dots, \beta_{L,j}^{(r,i)}\right)^T \sim t_{2\tilde{a}_\varsigma}\left(\widetilde{\boldsymbol{\beta}}_j^{(r,i)}, \frac{\tilde{b}_\varsigma}{\tilde{a}_\varsigma}\boldsymbol{\Sigma}_{\beta,j}^{(r,i)}\right), \qquad \varsigma_j^{-2(r,i)} \sim Ga\left(\tilde{a}_\varsigma, \tilde{b}_\varsigma\right),$$

where $\boldsymbol{\Sigma}_{\beta,j}^{(r,i)} = (\boldsymbol{X}_j^{(r,i)T}\boldsymbol{X}_j^{(r,i)} + \boldsymbol{I}_L)^{-1}$, $\widetilde{\boldsymbol{\beta}}_j^{(r,i)} = \boldsymbol{\Sigma}_{\beta,j}^{(r,i)}\boldsymbol{X}_j^{(r,i)T}\boldsymbol{\eta}_j^{(r,i)}$, $\tilde{a}_\varsigma = a_\varsigma + \frac{T-1}{2}$, $\tilde{b}_\varsigma = b_\varsigma + \frac{1}{2}\left(\boldsymbol{\eta}_j^{(r,i)T}\boldsymbol{\eta}_j^{(r,i)} - \widetilde{\boldsymbol{\beta}}_j^{(r,i)T}(\boldsymbol{\Sigma}_{\beta,j}^{(r,i)})^{-1}\,\widetilde{\boldsymbol{\beta}}_j^{(r,i)}\right)$ and a p- dimensional $t_\nu(\boldsymbol{x}; \boldsymbol{\mu}, \boldsymbol{\Sigma})$ denotes the multivariate central t distribution with location vector $\boldsymbol{\mu}$ and scale matrix $\boldsymbol{\Sigma}$ with pdf $\frac{\Gamma\left(\frac{\nu+p}{2}\right)}{\Gamma\left(\frac{\nu}{2}\right)(\nu\pi)^{\frac{p}{2}}|\boldsymbol{\Sigma}|^{\frac{1}{2}}}\left\{1 + \frac{1}{\nu}(\boldsymbol{x}-\boldsymbol{\mu})^T\boldsymbol{\Sigma}^{-1}(\boldsymbol{x}-\boldsymbol{\mu})\right\}^{-\frac{\nu+p}{2}}$.

Step 3 Sample the latent variables for updating $\boldsymbol{\Omega}$:

(i) Calculate the autocorrelation corrected Gaussian values $\hat{Z}_{t,j}^{(r,i)} = \frac{1}{\varsigma_j^{(r,i)}}\left(\tilde{Z}_{t,j}^{(r,i)} - \sum_{t'=1}^{L}\beta_{t',j}^{(r,i)}\tilde{Z}_{t-t',j}^{(r,i)}\right)$ and subsequently obtain $\boldsymbol{\Xi}^{(r,i)} = \widehat{\boldsymbol{Z}}^{(r,i)}\boldsymbol{R}_\Omega^{\frac{1}{2}}$ where $\widehat{\boldsymbol{Z}}^{(r,i)} = \left(\left(\hat{Z}_{t,j}^{(r,i)}\right)\right)$.

(ii) Construct the $NTR \times d$ matrix $\boldsymbol{W}$ by concatenating the $\boldsymbol{\Xi}^{(r,i)}$ matrices across all $r = 1, \dots, R$ and $i = 1, \dots, N$.

(iii) For notational convenience, we let $\widetilde{N} = NTR$ and define $\boldsymbol{W}_i = \left(W_{i,1}, \dots, W_{i,d}\right)^T$. Generate q-dimensional vectors $\boldsymbol{u}_1, \dots, \boldsymbol{u}_{\widetilde{N}} \overset{iid}{\sim} \mathrm{N}_q(\boldsymbol{0}, \boldsymbol{P})$ with $\boldsymbol{P} = \left(\boldsymbol{I}_q + \boldsymbol{\Lambda}^T\boldsymbol{\Delta}^{-1}\boldsymbol{\Lambda}\right)$ independently from $\boldsymbol{W}_{1:\widetilde{N}}$ and let $\boldsymbol{\vartheta}_i = \boldsymbol{W}_i + \boldsymbol{\Delta}^{-1}\boldsymbol{\Lambda}\boldsymbol{P}^{-1}\boldsymbol{u}_i$.

Step 4 Update $\boldsymbol{\Lambda}$: We have $\boldsymbol{u}_i = \sum_{r=1}^{d}\boldsymbol{\lambda}_r\vartheta_{r,i} + \boldsymbol{\varepsilon}_i$, where $\boldsymbol{\lambda}_r = \left(\lambda_{r,1}, \dots, \lambda_{r,q}\right)$ is the r-th row of $\boldsymbol{\Lambda}$ and $\boldsymbol{\vartheta}_i = \left(\vartheta_{1,i}, \dots, \vartheta_{d,i}\right)^T$. Define $\boldsymbol{u}_i^{(j)} = \boldsymbol{u}_i - \sum_{r \ne j}\boldsymbol{\lambda}_r\vartheta_{r,i}$. Then $\boldsymbol{u}_i^{(j)} = \boldsymbol{\lambda}_j\vartheta_{j,i} + \boldsymbol{\varepsilon}_i$. Conditioned on $\boldsymbol{u}_i^{(j)}$, $\boldsymbol{\vartheta}_i$ and the associated hyper-parameters, $\boldsymbol{\lambda}_j$'s can be updated sequentially for $j = 1, \dots, d$ from the distribution

$$\boldsymbol{\lambda}_j \sim \mathrm{N_q}\left\{\left(\boldsymbol{D}_j^{-1} + \left\|\boldsymbol{\vartheta}^{(j)}\right\|^2\boldsymbol{I}_q\right)^{-1}\boldsymbol{m}_j, \left(\boldsymbol{D}_j^{-1} + \left\|\boldsymbol{\vartheta}^{(j)}\right\|^2\boldsymbol{I}_q\right)^{-1}\right\},$$

where $\boldsymbol{D}_j = \tau^2\mathrm{diag}\left(\psi_{j,1}\phi_{j,1}^2, \dots, \psi_{j,q}\phi_{j,q}^2\right)$, $\boldsymbol{\vartheta}^{(j)} = \left(\vartheta_{j,1}, \dots, \vartheta_{j,N}\right)^T$ and $\boldsymbol{m}_j = \sum_{i=1}^{\widetilde{N}}\vartheta_{j,i}\boldsymbol{u}_i^{(j)}$.

Step 5 Update $\boldsymbol{\Delta}$ and α: Sample the δ_j^2's through the following steps.

(i) Let $d_{r,-j} = \sum_{l \neq j} 1(\varsigma_l = r)$ and $\boldsymbol{\vartheta}^{(-j)}$ to be the collection of all $\boldsymbol{\vartheta}^{(l)}$'s, $l = 1, \ldots, d$, excluding $\boldsymbol{\vartheta}^{(j)}$ and $1(\cdot)$ be the indicator function. For $j = 1, \ldots, d$, sample the cluster indicators sequentially from the distribution

$$\Pr(\varsigma_j = r) \propto \begin{cases} d_{r,-j} \int \mathrm{N}\left(\boldsymbol{\vartheta}^{(j)}; 0, \delta_r^{-2}\right) \mathrm{d}G_0\left(\delta_r^2 | \boldsymbol{\vartheta}^{(-j)}\right) \text{ for } r \in \{\varsigma_l\}_{l \neq j}; \\ \alpha \int \mathrm{N}\left(\boldsymbol{\vartheta}^{(j)}; 0, \delta_r^{-2}\right) \mathrm{d}G_0(\delta_r^2) \text{ for } r \neq \varsigma_l \text{ for all } l \neq j. \end{cases}$$

The above integrals are analytically available and involves the density of a multivariate central Student's t-distribution for $G_0 = \mathrm{Ga}(a_\delta, b_\delta)$.

(ii) Let the unique values in $\varsigma_{1:d}$ be $\{1, \cdots, k\}$. For $r = 1, \ldots, k$, set $d_r = \sum_j 1(\varsigma_j = r)$ and $V_r = \sum_{j:\varsigma_j = r} \left\| \vartheta^{(j)} \right\|^2$, and independently sample $\delta_r^2 \sim \mathrm{Ga}(a_\delta + N d_r/2, b_\delta + V_r/2)$.

(iii) Set $\delta_j^2 = \delta_{\varsigma_j}^2$.

(iv) Following (West, 1992), first generate $\varphi \sim \mathrm{Beta}(\alpha + 1, d)$, evaluate $\pi/(1 - \pi) = (a_\alpha + k - 1)/\{d(b_\alpha - log\varphi)\}$ and then generate

$$\alpha | \varphi, k \sim \begin{cases} \mathrm{Ga}(\alpha + k, b_\alpha - log\varphi) \text{ with probability } \pi; \\ \mathrm{Ga}(\alpha + k - 1, b_\alpha - log\varphi) \text{ with probability} 1 - \pi. \end{cases}$$

Step 6 Update Dirichlet-Laplace hyperparameters: Sample the hyper-parameters in the priors on $\boldsymbol{\Lambda}$ through the following steps.

(i) For $j = 1, \ldots, d$ and $h = 1, \ldots q$ sample $\Phi_{j,h}$ independently from an inverse-Gaussian distribution $\mathrm{iG}\left(\tau\phi_{j,h}/|\lambda_{j,h}|, 1\right)$ and set $\psi_{j,h} = 1/\Phi_{j,h}$.

(ii) Sample the full conditional posterior distribution of τ from a generalized inverse Gaussian $\mathrm{giG}\left\{dq(1 - a), 2b, 2\sum_{j,h} |\lambda_{j,h}|/\phi_{j,h}\right\}$ distribution.

(iii) Draw $T_{j,h}$ independently with $T_{j,h} \sim \mathrm{giG}\left(a - 1, 1, 2|\lambda_{j,h}|\right)$ and set $\phi_{j,h} = T_{j,h}/T$ with $T = \sum_{j,h} T_{j,h}$.

Remark: In the sampler described above, the dependence across the ROIs via the shared correlation matrix $\boldsymbol{R}_\Omega$ is ignored while updating the mixture model parameters $\left\{\pi_{h,j}^{(r,i)}, \mu_{h,j}^{(r,i)}, \sigma_{h,j}^{2(r,i)}\right\}_{h=1}^{K}$ as well as the AR model parameters $\left(\boldsymbol{\beta}_j, \varsigma_j^{2(r,i)}\right)$ across $j = 1, \ldots, d$. Such strategies significantly reduce computational complexity (Grazian & Liseo, 2015). As this model implementation strategy yielded highly consistent results in thorough cross-validation as well as in simulation studies reported later in Section S1.6 of the supplement, we conjecture that this

strategy has negligible deviance from an exact but numerically significantly more expensive MCMC sampler.

S1.3 Choice of Hyperparameters

Autoregressive model hyperparameters: We let $\nu_\beta = 1$ and set $(a_\varsigma, b_\varsigma)$ such that *a priori* $E\left(\varsigma_j^{2(r,i)}\right) = 1$ and $\text{Var}\left(\varsigma_j^{2(r,i)}\right) = 5$ for all r, i, j to ensure weakly informative priors. Leveraging the idea of '*Bayesian Ockham razor'* (Jefferys & Berger, 1992), we over-specified the order or lag of the autoregressive models L and considered a mean zero conditionally Gaussian prior on the AR model parameters $\beta_{1,j}^{(r,i)}, \dots, \beta_{L,j}^{(r,i)}$ inducing default shrinkage towards zeroes. The mean zero prior adaptively learns from the data to shrink the redundant β parameters (van Erp et al., 2019). In all our analyses, we set the order of the autoregressive models $L = 5$.

Mixture model hyperparameters: Regarding the Dirichlet prior's concentration parameter, we let $\alpha_\pi = 1$. Regarding the normal-inverse gamma prior on the location-scale parameters of the mixture components, we let $\mu_0 = 0$, $\nu_0 = 0.01$, $a_0 = 250$ and $b_0 = 20$. Such hyperparameter choices imply $E\left(\mu_{h,j}^{(r,i)}\right) = 0$ and $E\left(\sigma_{h,j}^{2(r,i)}\right) \approx 0$ for all r, i, j, h. Based on previous experience on Gaussian mixture models, we observed that such choices favor a larger number of occupied clusters in the mixture model a posteriori, allowing for a more flexible fit. We set number of mixture components $K = 20$.

Precision factor analysis model hyperparameters: In this paper we set the column-dimension (q) of Λ $q = d$ where d is the row-dimension of Λ, i.e., the number of ROIs of interest. Since the number of ROIs is in the order of tens in this paper, we take q=d to have a full-rank model.

Following the suggestions of (Bhattacharya et al. 2015; Chandra et al. 2021) we let $a = 0.5$ and $b = 2$ as the hyperparameters of the two- parameter Dirichlet-Laplace prior on Λ. For the prior on the residual variances and the Dirichlet process concentration parameter, we set $a_\delta = b_\delta = a_\alpha = b_\alpha = 0.10$ implying weakly informative priors.

S1.4 Graph Selection

Typical of Bayesian continuous shrinkage priors, exact zero estimates are not obtained even for the insignificant off-diagonal elements of $\mathbf{\Omega}$ for finite sample sizes. This is an artifact of continuous shrinkage priors, since the probabilities of exact zeroes are *almost surely* zero although the posterior probabilities of arbitrary sets around zeroes are very high.

We address the issue of non-zero edge selection through a multiple hypothesis testing based approach. For $i = 1, \dots, d, j = i + 1, \dots, d$ and some $\epsilon > 0$, we consider testing

$$H_{0,i,j}: |\rho_{i,j}| \leq \epsilon \text{ versus } H_{1,i,j}: |\rho_{i,j}| > \epsilon,$$

where $\rho_{i,j}$ is the (i, j)-th element of $diag(\mathbf{\Omega})^{-\frac{1}{2}} \mathbf{\Omega}\, diag(\mathbf{\Omega})^{-\frac{1}{2}}$, the partial correlation matrix derived from $\mathbf{\Omega}$. Here we follow (Berger, 1985) (Chapter 4, pp. 148) in replacing the point nulls $H_{0,i,j}: \rho_{i,j} = 0$

by reasonable interval nulls $H_{0,i,j}: |\rho_{i,j}| \leq \epsilon$. If $H_{0,i,j}$ is rejected in favor of $H_{1,i,j}$, we conclude that there is an edge between nodes i and j. Next, we define $d_{i,j} = 1\{\Pi(H_{1,i,j}|Y) > \beta\}$ as the decision rule which controls the posterior FDR defined as

$$FDR_Y = \frac{\sum_{i,j} d_{i,j} \Pi(H_{0,i,j}|Y)}{\max(\sum_{i,j} d_{i,j}, 1)},$$

at the level $1 - \beta$. Importantly, the decision rule also incurs the lowest false non-discovery rate (Müller et al., 2004). For a fixed β, the FDR_Y depends on the choice of ϵ. To obtain the optimal ϵ, we compute the FDR_Y's on a grid of ϵ values in $(0,1)$ and then set $\epsilon = \arg\inf_{\epsilon'} FDR_Y(\epsilon') \leq 1 - \beta$. In all applications in this paper, we control FDR_Y at the 0.10 level of significance.

S1.5 Control ROI graph

To assess whether our ARMGCGM approach is sensitive to connectivity differences across brain networks, we conducted an analysis that included four control regions (two per hemisphere): pericalcarine cortex and superior frontal gyrus. Pericalcarine cortex is the anatomical location of primary visual cortex, while superior frontal gyrus was selected due to its lack of association with auditory processing in Neurosynth (https://neurosynth.org/analyses/terms/auditory/). Each of these control regions is associated with cortical networks that are distinct from networks involved in auditory processing (Yeo et al., 2011).

Because partial correlations are influenced by the nodes that are included in the graph, specific edges may change depending on the makeup of the graph. However, we found that connectivity between auditory regions did not differ notably in this graph that includes control regions. Additionally, the strongest partial correlations of each control region were with its contralateral homolog. These results were consistent between data splits (by acquisition scheme). These results suggest that partial correlations using our ARMGCGM are specific to their individual subnetworks.

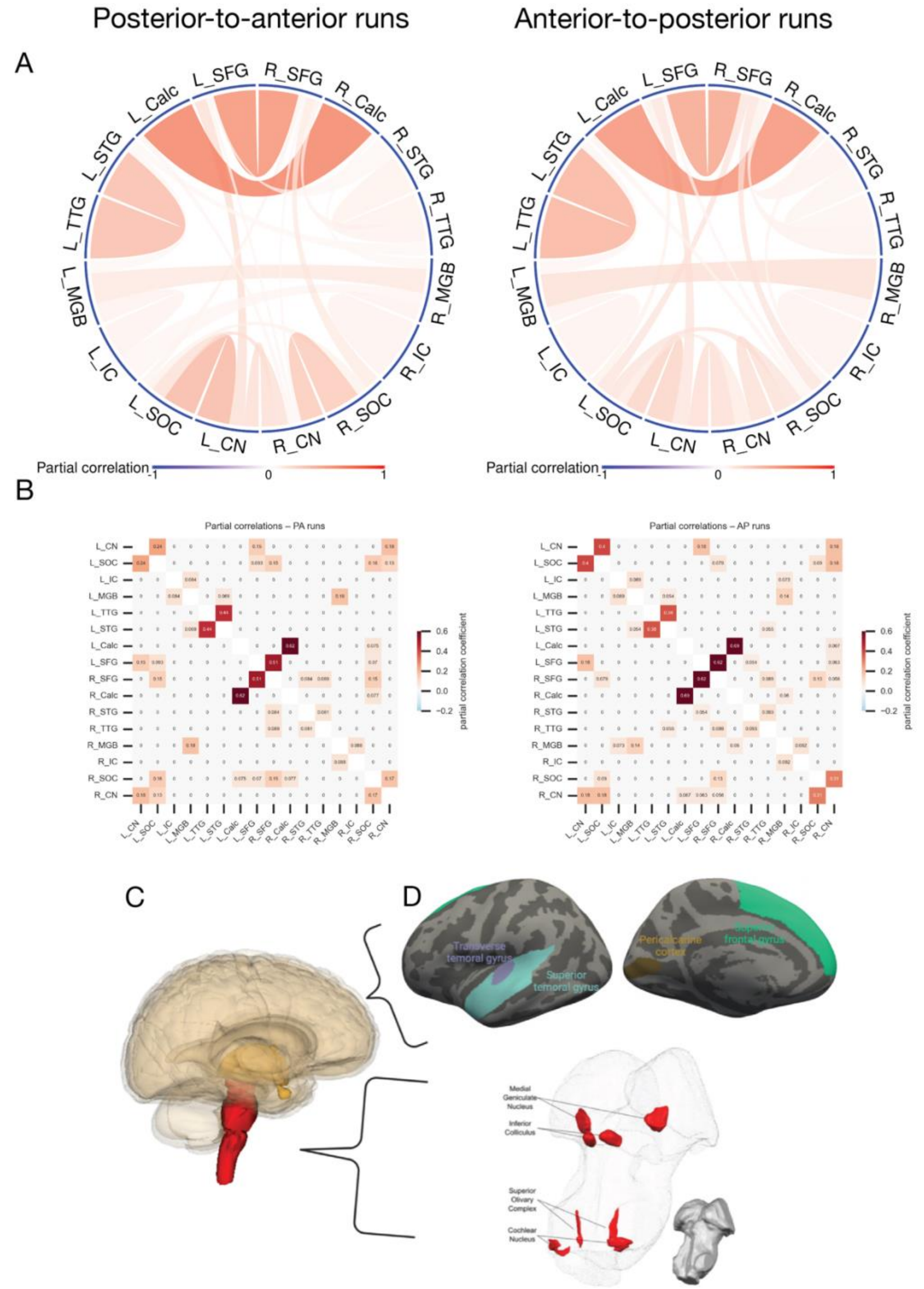

Figure S.3. A: Partial correlation connectivity in data acquired with posterior-to-anterior (PA; left) and anterior-to-posterior (AP; right) phase-encoding directions using the ARMGCGM approach. Positive (negative) associations are represented by red (blue) links, their opacities being proportional to the corresponding association strengths. The link widths are inversely proportional to the number of edges associated with the corresponding nodes. B: The same results as (A), viewed as adjacency matrices (left = PA runs; right = AP runs). C: 3-dimensional view of the human brain with the brainstem highlighted in red. C: regions of interest from which functional timeseries were extracted. Top: cortical regions from FreeSurfer's DKT atlas. Bottom: subcortical auditory regions (Sitek et *al.*, 2019).

S1.6 Data and analysis workflow

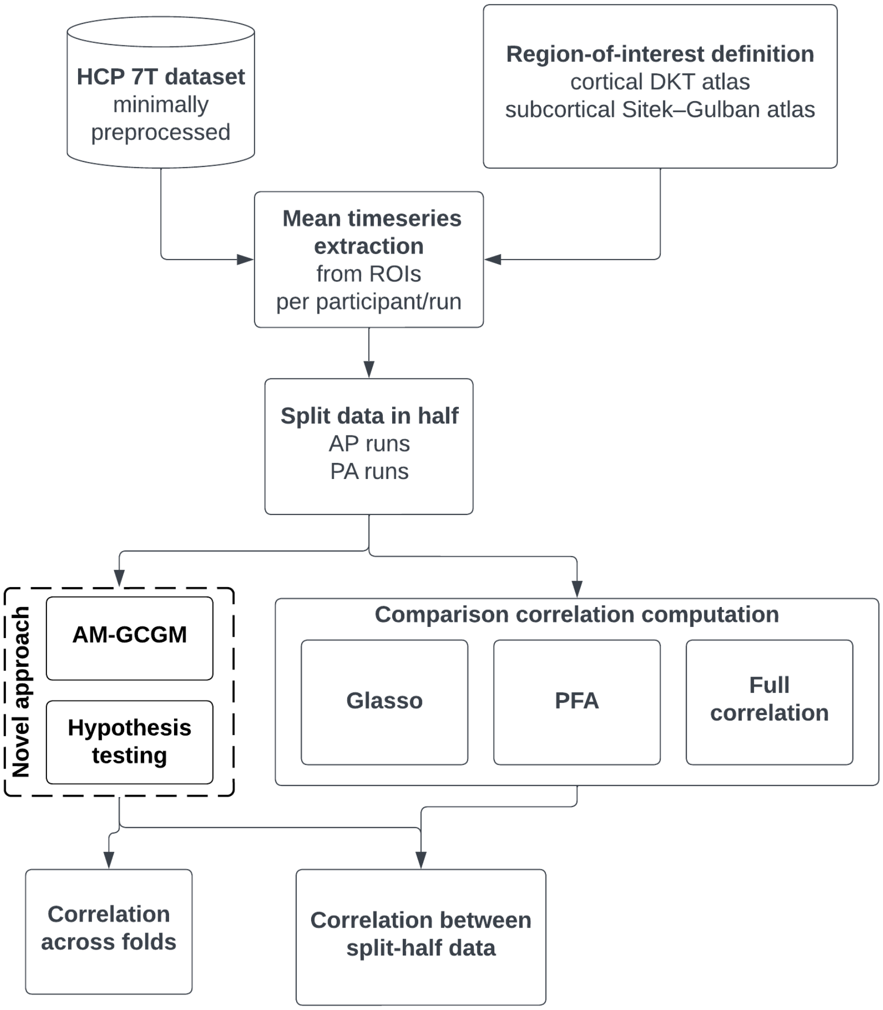

Figure S.4. High-level diagram of materials and methods implemented in this manuscript.

S1.7 Simulation Study

In this section we do simulation studies to check whether our proposed autoregressive matrix-Gaussian copula graphical model (ARMGCGM) can recover the conditional dependence structure from non-Gaussian data with autocorrelation.

True Simulation Settings: To create realistic simulation setups, we use predictive simulation to emulate the fMRI data, taking advantage of the observed $Y_{t,j}^{(r,i)}$'s as outlined below. We repeat the simulations for both phase-encoding schemes.

Step 1. We sample $\boldsymbol{\Omega}$ from G-Wishart distribution using the `BDgraph R` package (Mohammadi & Wit, 2019) using the recovered adjacency and precision matrices reported in Figure 2 of the main paper. Then we set the correlation matrix $\boldsymbol{R}_{\Omega} = \boldsymbol{\Psi}^{-\frac{1}{2}}\boldsymbol{\Omega}^{-1}\boldsymbol{\Psi}^{-\frac{1}{2}}$ with $\boldsymbol{\Psi} = \mathrm{diag}(\boldsymbol{\Omega}^{-1})$.

Step 2. For $r = 1, \ldots, R,\ i = 1, \ldots, N$ and $t = 1, \ldots, T$, we sample

$$\left(\xi_{t,1}^{(r,i)}, \ldots, \xi_{t,d}^{(r,i)}\right)^T \overset{iid}{\sim} \mathrm{N}_d(0, \boldsymbol{R}_\Omega).$$

In accordance with the observed data, we set $R = 2, N = 106$ and $T = 850$.

Step 3. Let $\left\{Y_{t,j}^{(r,i)}\right\}_{t=1}^{T}$ be the observed fMRI timeseries as described in Section 2.3 of the main paper. For $r = 1, \ldots, R,\ i = 1, \ldots, N$ and $j = 1, \ldots, d$,

i. we fit a Gaussian mixture model with 5 mixture components on $\{Y_{1,j}^{(r,i)}, \ldots, Y_{T,j}^{(r,i)}\}$ using the `mclust R` package (Scrucca et al., 2023) . Let $\hat{F}_j^{(r,i)}(\cdot)$ denote the CDF of the recovered mixture model;

ii. we calculate $Z_{t,j}^{(r,i)} = \Phi^{-1}\left\{\hat{F}_j^{(r,i)}\left(Y_{t,j}^{(r,i)}\right)\right\}$ where $\Phi(\cdot)$ denotes the CDF of a standard normal distribution;

iii. we fit an autoregressive (AR) model of lag 5 on $\{Z_{t,j}^{(r,i)}\}_{t=1}^{T}$ in `R` to recover the AR model parameters. Using the recovered parameters we transform the sampled iid sequence $\{\xi_{t,j}^{(r,i)}\}_{t=1}^{T}$ to an $AR(5)$ timeseries $\{\tilde{Z}_{t,j}^{(r,i)}\}_{t=1}^{T}$ using the `stats::filter` function in `R`.

Step 4. For all $t = 1, \ldots, T$, we obtain $\tilde{Y}_{t,j}^{(r,i)}$ by setting

$$\tilde{Y}_{t,j}^{(r,i)} = arg\ min_x \{\hat{F}_j^{(r,i)}(x) - \Phi\left(\tilde{Z}_{t,j}^{(r,i)}\right)\}^2.$$

Solution of the above equation is not available analytically. We used the L-BFGS-B method (Byrd et al., 1995) as implemented in the `stats::optim` function in `R`.

In Step 1 we sampled a correlation matrix $\boldsymbol{R}_\Omega$ that is similar to the one recovered from the observed fMRI data by ARMGCGM. In Step 2 we sampled iid d-variate mean 0 normal data having correlation structure $\boldsymbol{R}_\Omega$. In Step 3(iii) we induced an autocorrelation structure into the sampled iid sequence $\{\xi_{t,j}^{(r,i)}\}_{t=1}^{T}$ to obtain $\{\tilde{Z}_{t,j}^{(r,i)}\}_{t=1}^{T}$, which reflects the structure initially observed in the corresponding fMRI data. Finally in Step 4, we transformed the Gaussian-distributed $\{\tilde{Z}_{t,j}^{(r,i)}\}_{t=1}^{T}$ into non-Gaussian distributed $\{\tilde{Y}_{t,j}^{(r,i)}\}_{t=1}^{T}$, thereby emulating their respective observed fMRI data.

Simulation results: We report the results of the predictive simulations in Figure S.5; the top and bottom panels correspond to the predictive simulations in the posterior-to-anterior true and anterior-to-posterior acquisition schemes, respectively. In each scheme, true and estimated functional connectivity graphs are reported from left to right. The simulation studies indicate the ARMGCGM can indeed recover the underlying functional connectivity graphs of fMRI data.

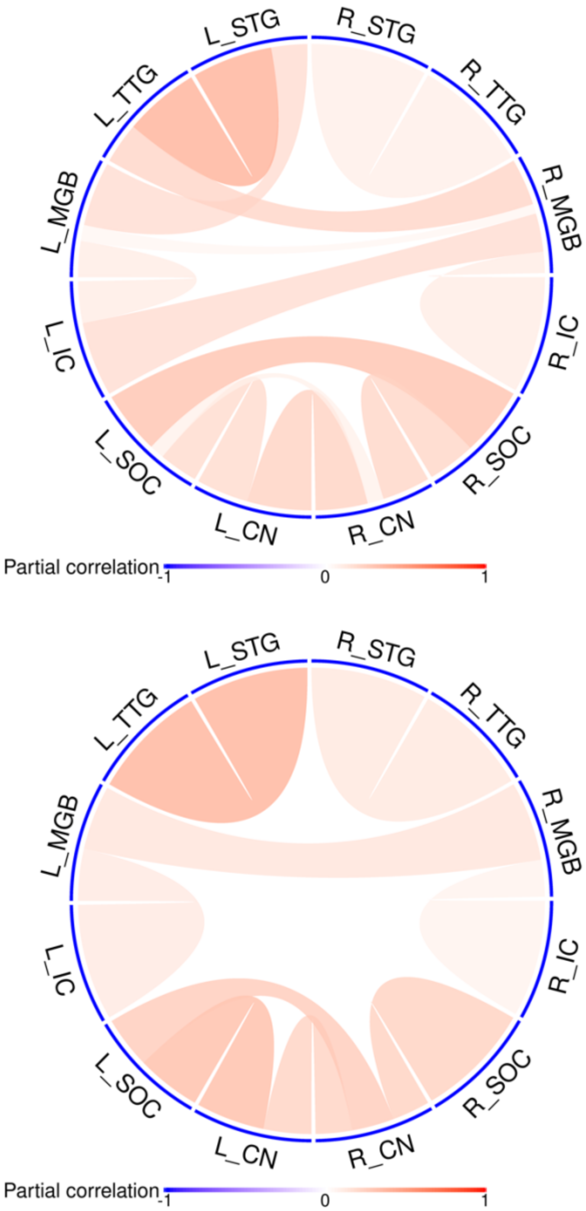

Figure S.5. Results of the simulation studies: In the top panel we report results for predictive simulations in the posterior-to-anterior acquisition scheme; we plot the true and recovered functional connectivity graphs from left to right, respectively. The same is reported for the anterior-to-posterior acquisition scheme in the bottom panel.

References

Berger, J. O. (1985). Bayesian Analysis. In *Statistical decision theory and bayesian analysis* (pp. 118–307). Springer New York. https://doi.org/10.1007/978-1-4757-4286-2_4

Byrd, R. H., Lu, P., Nocedal, J., & Zhu, C. (1995). A Limited Memory Algorithm for Bound Constrained Optimization. *SIAM Journal on Scientific Computing*, *16*(5), 1190–1208. https://doi.org/10.1137/0916069

Grazian, C., & Liseo, B. (2015). Approximate Bayesian Computation for Copula Estimation. *Dep. of Statistical Sciences "Paolo Fortunati", Università Di Bologna*. https://doi.org/10.6092/issn.1973-2201/5827

Jefferys, W. H., & Berger, J. O. (1992). Ockham's Razor and Bayesian Analysis. *JSTOR*.

Mohammadi, R., & Wit, E. C. (2019). bdgraph : an *r* package for bayesian structure learning in graphical models. *Journal of Statistical Software*, *89*(3). https://doi.org/10.18637/jss.v089.i03

Müller, P., Parmigiani, G., Robert, C., & Rousseau, J. (2004). Optimal sample size for multiple testing. *Journal of the American Statistical Association*, *99*(468), 990–1001. https://doi.org/10.1198/016214504000001646

Scrucca, L., Fraley, C., Murphy, T. B., & Raftery, A. E. (2023). Model-Based Density Estimation. In *Model-Based Clustering, Classification, and Density Estimation Using mclust in R* (pp. 129–152). Chapman and Hall/CRC. https://doi.org/10.1201/9781003277965-5

van Erp, S., Oberski, D. L., & Mulder, J. (2019). Shrinkage priors for Bayesian penalized regression. *Journal of Mathematical Psychology*, *89*, 31–50. https://doi.org/10.1016/j.jmp.2018.12.004

West, B. (1992). Hyperparameter estimation in Dirichlet process mixture models. *Undefined*.

Yeo, B. T. T., Krienen, F. M., Sepulcre, J., Sabuncu, M. R., Lashkari, D., Hollinshead, M., Roffman, J. L., Smoller, J. W., Zöllei, L., Polimeni, J. R., Fischl, B., Liu, H., & Buckner, R. L. (2011). The organization of the human cerebral cortex estimated by intrinsic functional connectivity. *Journal of Neurophysiology*, *106*(3), 1125–1165. https://doi.org/10.1152/jn.00338.2011
